# A metastasis map of human cancer cell lines

**DOI:** 10.1038/s41586-020-2969-2

**Published:** 2020-12-09

**Authors:** Xin Jin, Zelalem Demere, Karthik Nair, Ahmed Ali, Gino B. Ferraro, Ted Natoli, Amy Deik, Lia Petronio, Andrew A. Tang, Cong Zhu, Li Wang, Danny Rosenberg, Vamsi Mangena, Jennifer Roth, Kwanghun Chung, Rakesh K. Jain, Clary B. Clish, Matthew G. Vander Heiden, Todd R. Golub

**Affiliations:** 1 Broad Institute of MIT and Harvard, Cambridge, MA USA; 2 Koch Institute for Integrative Cancer Research, Department of Biology, Massachusetts Institute of Technology, Cambridge, MA USA; 3 Edwin L. Steele Laboratories, Department of Radiation Oncology, Massachusetts General Hospital, Boston, MA USA; 4 Institute for Medical Engineering and Science, Picower Institute for Learning and Memory, Department of Chemical Engineering, Massachusetts Institute of Technology, Cambridge, MA USA; 5 Harvard Medical School, Boston, MA USA; 6 Dana-Farber Cancer Institute, Boston, MA USA

**Keywords:** Breast cancer, Cancer genomics, Cancer models, Metastasis

## Abstract

Most deaths from cancer are explained by metastasis, and yet large-scale metastasis research has been impractical owing to the complexity of in vivo models. Here we introduce an in vivo barcoding strategy that is capable of determining the metastatic potential of human cancer cell lines in mouse xenografts at scale. We validated the robustness, scalability and reproducibility of the method and applied it to 500 cell lines^1,2^ spanning 21 types of solid tumour. We created a first-generation metastasis map (MetMap) that reveals organ-specific patterns of metastasis, enabling these patterns to be associated with clinical and genomic features. We demonstrate the utility of MetMap by investigating the molecular basis of breast cancers capable of metastasizing to the brain—a principal cause of death in patients with this type of cancer. Breast cancers capable of metastasizing to the brain showed evidence of altered lipid metabolism. Perturbation of lipid metabolism in these cells curbed brain metastasis development, suggesting a therapeutic strategy to combat the disease and demonstrating the utility of MetMap as a resource to support metastasis research.

## Abstract (web summary)

A method in which pooled barcoded human cancer cell lines are injected into a mouse xenograft model enables simultaneous mapping of the metastatic potential of multiple cell lines, and shows that breast cancer cells that metastasize to the brain have altered lipid metabolism.

## Main

Human cancer cell lines have been a driving force in cancer research, leading to the discovery of oncogenic mechanisms and therapeutic targets^1–4^. However, large-scale characterization of cell lines has been limited to rudimentary readouts such as viability in cell culture, because more complex phenotypes—such as behaviours in vivo—have not been tractable at scale. By contrast, most studies of metastasis rely on only a small number of experimental models^5–9^, thereby making it difficult to extrapolate findings to genetically diverse human tumours^10^.

Ideally, it would be possible to construct a map of organ-specific metastatic potential of hundreds of human cancer cell lines using xenograft models, so that the molecular features of the cell lines could be related to their ability to survive and proliferate in organ-specific microenvironments. However, the prospect of in vivo testing of each cell line individually is unattractive, because it is labour-intensive and expensive, as well as because of the difficulty in sufficiently controlling for variability between animal experiments. We proposed that if cell lines were labelled with molecular barcodes and injected into recipient mice as a pool, internally controlled, metastatic potential could be assessed in a highly scalable manner.

## Pilot study with breast cancer

**Fig. 1:**
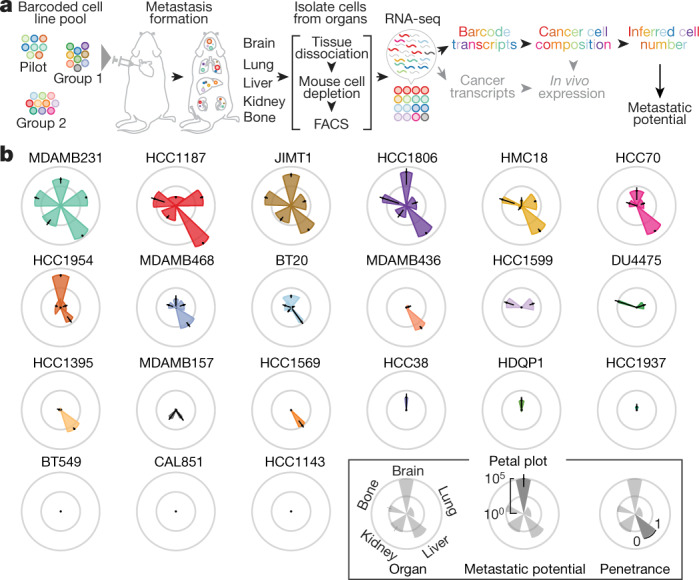
Scalable in vivo metastatic potential mapping with barcoded cell line pools. **a**, A schematic of the experiment determining the feasibility of in vivo metastatic potential profiling using barcoded cell line pools. Barcode abundance reflecting cancer cell compositions was determined by RNA-seq, and the cell number of each cell line was inferred by cancer cell composition and total cancer cell counts isolated from the target organ. **b**, Petal plots displaying the metastatic patterns of 21 basal-like breast cancer cell lines. Petal length represents metastatic potential, quantifying the mean of inferred cancer cell numbers detected from the target organs. Data are mean ± 95% confidence interval. Petal width shows penetrance, quantifying percentage of mice detected with the cell line. Source data.

**Extended Data Fig. 1:**
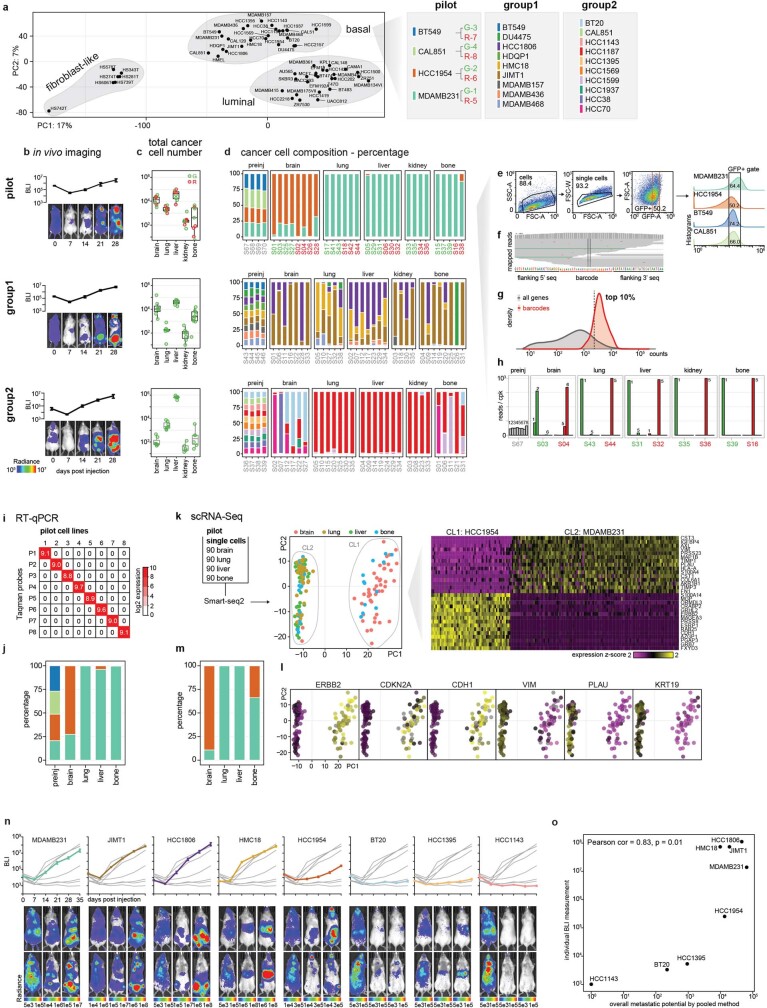
An in vivo barcoding approach to establish multiplexed cancer metastasis xenografts and validation using orthogonal assays. **a**, Principal component analysis (PCA) of transcriptomic expression of the breast cancer collection from CCLE, and the pooling schemes focusing on basal-like breast cancer. G, GFP; R, mCherry. The linked numbers indicate the labelling barcodes. **b**, Real-time BLI monitoring of the overall metastasis progression from pilot, group1, group2 cell line pools. Data are mean ± s.e.m. *n* = 5 (pilot), *n* = 8 (group1), *n* = 7 (group2) mice. **c**, Total cancer cell numbers isolated by FACS from each target organ from pilot, group1, group2 pools. Each dot represents an animal. Box plots display quartiles of the data. **d**, Cancer cell composition of metastases from different organs as determined by barcode abundance from pilot, group1, group2 pools. pilot: G portion samples are highlighted in green, R portion samples are highlighted in red. preinj, pre-injected population. Data **c**, **d** were used to quantify the metastatic potential presented in Fig. 1b. **e**, An example of the gating strategy to isolate GFP+ barcoded cancer cells for the pilot pool. Infected cell lines expressed GFP at different levels as shown in the histogram, and a fixed gate was used to enrich cells with closer expression level. Numbers correspond to cell percentages within the gate. **f**, An example of barcode mapping result visualized by Integrative Genomics Viewer (IGV). **g**, Distribution of the barcode read counts versus all gene transcript counts. Barcodes are among the top 10% highly expressed genes, allowing robust quantification. **h**, An example of barcode read quantification in the pre-injected and metastasis samples from pilot pool. Barcodes are listed as in **a**. cpk, counts per thousand. **i**, Taqman assay on in vitro cultured barcoded cell lines from the pilot pool. The signal is very specific to each barcode and there is no cross detection. **j**, Quantification of barcode abundance and cancer cell composition using the Taqman RT–qPCR assay in the pre-injected and metastasis samples from the pilot pool. The results agree with barcode quantification from bulk RNA-Seq (Extended Data Fig. 1d). **k**, Single cell RNA-Seq of metastases from different organs from the pilot pool. Single cancer cells isolated from each organ were sorted into 96-well plates, with 90 cells per plate (rest 6 wells for positive and negative controls), and subjected to Smart-Seq2. PCA revealed that PC1 maximally separated the cancer cells into 2 clusters (CLs), with CL1 enriched in cells isolated from brain, and CL2 enriched in cells isolated from lung, liver and bone. Heat map on the right shows gene expression that associates with PC1 and clustering of cells. Based on marker expression, CL1 corresponds to HCC1954 (ERBB2+, CDH1+) and CL2 corresponds to MDAMB231 (CDKN2A loss, VIM+). **l**, Projection of marker gene expression on the PCA plot. **m**, Cancer cell composition based on single cell RNA-Seq data. The results agree with barcode quantification from bulk RNA-Seq (Extended Data Fig. 1d). **n**, Real-time BLI monitoring of metastasis progression of the 8 cell lines that were individually tested. Each plot highlights one of the 8 lines. Data are mean ± s.e.m. Each group contains 4 mice. **o**, Scatter plot showing the correlation of overall metastatic potential (5 organs combined) from pooled cell line experiments with whole body BLI of metastases measured individually. Pearson’s correlation coefficient and its test *P* value are presented. Source data

To test the feasibility and reliability of in vivo barcoding to monitor growth in different tissues in mice, we performed a pilot study using four breast cancer cell lines (Fig. 1a, Extended Data Fig. 1, Supplementary Note 1). Each cell line was engineered to express a unique 26-nucleotide barcode, together with luciferase for in vivo imaging and either GFP or mCherry to facilitate subsequent cell sorting and measurement of reproducibility within a single mouse (Extended Data Fig. 1a, Supplementary Table 1). The 8 barcoded lines were injected as a pool into the left ventricle of 5–6-week-old NOD-SCID-gamma (NSG) mice so as to focus our analysis on the ability of tumour cells to exit circulation and undergo expansion in distant organs. Bioluminescence imaging (BLI) revealed metastatic lesions throughout the body (Extended Data Fig. 1b). Five weeks after injection, brain, lung, liver, kidney and bone were collected, human tumour cells were isolated by fluorescence-activated cell sorting (FACS) using GFP or mCherry, and barcodes were quantified using RNA sequencing (RNA-seq) (Extended Data Fig. 1c–g). Whereas barcode abundances were similar pre-injection, some barcodes were enriched in specific organs (Extended Data Fig. 1h). Different cell lines exhibited distinct patterns of metastatic spread, but each cell line showed highly similar pattern of spread across multiple mice independent of whether GFP or mCherry versions were used, demonstrating the reproducibility of this pooled approach (Extended Data Fig. 1d). For example, HCC1954 was most strongly detected in brain, whereas extracranial metastases were dominated by MDAMB231. Barcodes quantified by bulk RNA-seq were independently validated by quantitative PCR with reverse transcription (RT–qPCR) and single-cell RNA-seq (Extended Data Fig. 1i–m, Supplementary Note 1).

Having validated the method, we next characterized the metastatic behaviours of all 21 basal-like breast cancer cell lines in the Cancer Cell Line Encyclopedia (CCLE) (Extended Data Fig. 1a–d). Basal-like breast cancers are known to have diverse metastatic abilities in patients^11^. Reflecting this diversity, the cell lines showed disparate metastatic patterns: pan-metastatic, metastatic preferentially to particular organs or not metastatic (Fig. 1b, Supplementary Table 2). Notably, one cell line (BT20) was detected in multiple organs, but at very low abundance in all of them, reflecting its ability to colonize but not expand. To validate the patterns of metastasis observed in the pooled in vivo system, we selected eight cell lines for individual characterization, and observed similar results from the pooled and individual screens (Extended Data Fig. 1n, o).

## A metastasis map of 500 human cancer cell lines

**Fig. 2:**
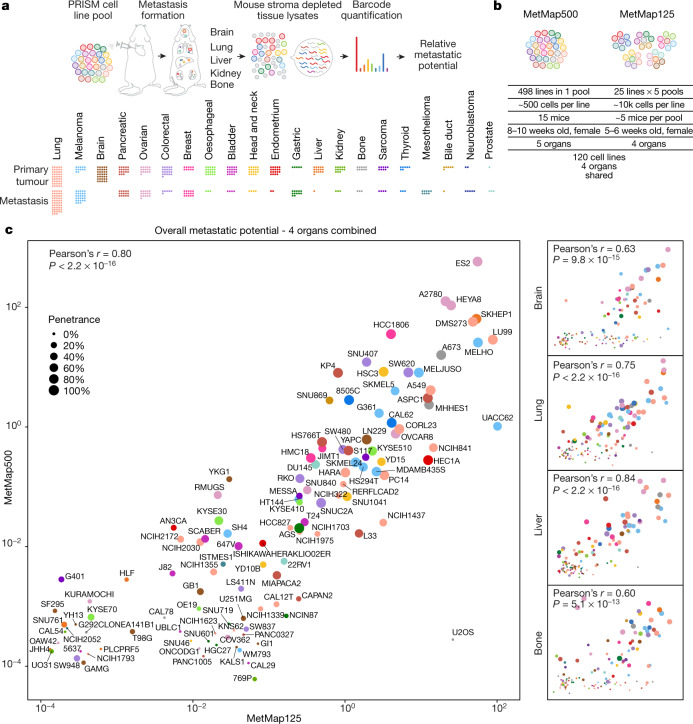
Drafting MetMap for 500 human cancer cell lines. **a**, A schematic of the workflow using pan-cancer PRISM cell line pools for high-throughput metastatic potential profiling. Relative metastatic potential was quantified by deep sequencing of PRISM barcode abundance from tissue. The cancer lineage distribution of the profiled 500 cancer cell lines is presented, with each dot representing a cell line, and showing whether the cell line was derived from primary tumour or metastasis. **b**, Comparison of experimental conditions between MetMap500 and MetMap125. **c**, Scatter plots showing overall and organ-specific metastatic potential as determined in MetMap500 and MetMap125. Strong correlation is observed between the two experiments. Each dot represents a cell line. Cancer lineage is colour-coded as in **a**. Source data.

**Extended Data Fig. 2:**
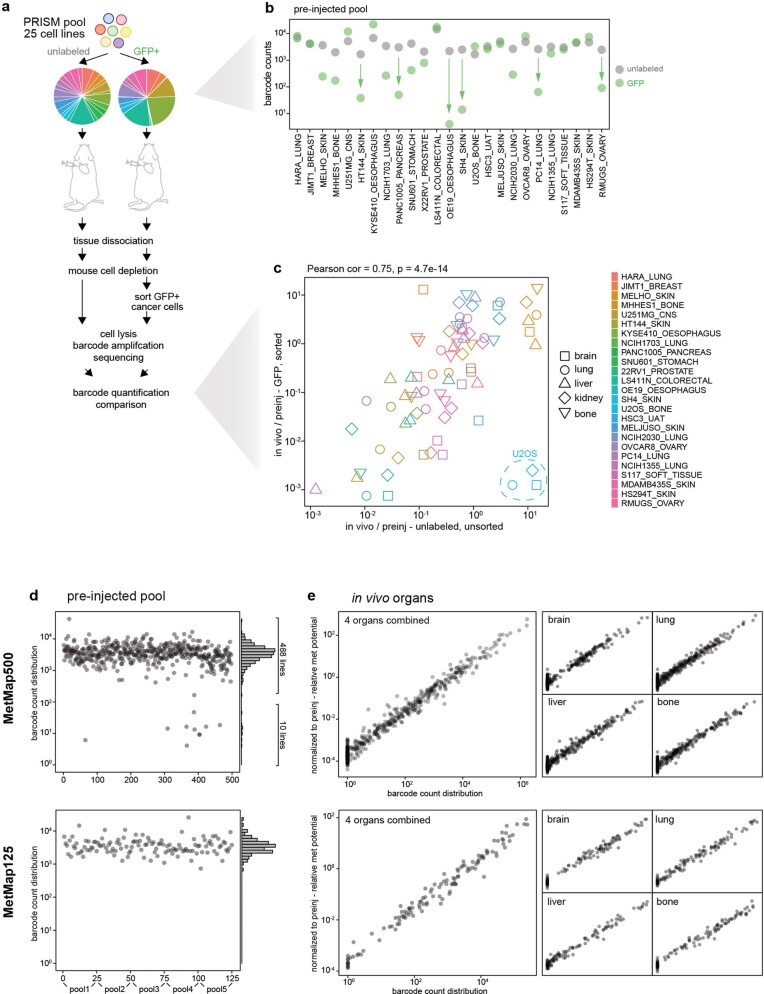
Using PRISM cell line pools for metastatic potential profiling. **a**, Optimizing the workflow of metastatic potential mapping using PRISM. A PRISM pool of 25 cell lines was used for testing the need of GFP labelling and cancer cell purification. The barcode abundance altered compared to the unlabelled population after GFP labelling as shown by the pie chart. **b**, A detailed line-by-line view of barcode abundance before and after GFP labelling. The unlabelled cell pool had more even distribution. Post labelling, several lines showed noticeable dropout, but all lines were detectable. **c**, Scatter plot comparing barcode enrichment after normalizing to the pre-injected input from the two experiments. Pearson’s correlation coefficient and its test *P* value are presented. Strong positive correlation is observed, with the exception of one cell line U2OS. **d**, Quality control of MetMap500 and MetMap125 datasets showing initial barcode abundance in the pre-injected populations. MetMap500, 1 large pool containing 498 cell lines was profiled, with 10 cell lines showing low initial abundance. These 10 cell lines were not detected in any in vivo sample, and were excluded from subsequent analysis. MetMap125, 5 pools of 25 cell lines were profiled separately and data were combined for analysis. **e**, Quality control of MetMap500 and MetMap125 datasets showing scatter plots of raw barcode abundance from in vivo organs versus the data normalized to the pre-injected input (in **d**). A strong linear relationship was observed. Source data

Having demonstrated its feasibility in breast cancer, we attempted to expand the mapping of metastatic potential to human cancer cell lines from diverse lineages. To facilitate higher-throughput profiling, we used cell lines barcoded for use with the PRISM method, which was developed for in vitro drug-sensitivity screening^12^. A simplified workflow enabled the quantitative detection of barcodes from crude tissue lysates without the need for FACS-based tumour cell purification (Extended Data Fig. 2, Supplementary Note 2). We applied this method to 503 cell lines spanning 21 lineages to develop a first-generation Metastasis Map (MetMap) (Fig. 2a). The data and interactive visualization are publicly accessible at https://pubs.broadinstitute.org/metmap.

**Extended Data Fig. 3:**
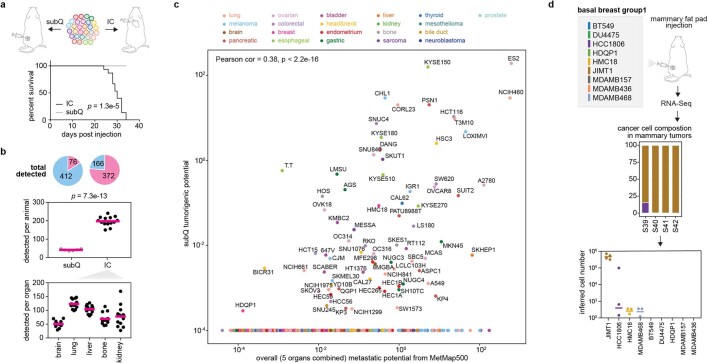
Subcutaneous injection of PRISM cell line pool. **a**, The same PRISM pool of 498 cell lines used for MetMap500 profiling was tested using subcutaneous injection on a cohort of 6 mice. Survival curves compare animal survival difference between subcutaneous and intracardiac (IC) injections, *P* value calculated using two-sided, log-rank test. **b**, Total numbers of cell lines detected in animals from the subcutaneous and IC injections. Detected lines are coloured in pink and non-detected lines are coloured in light-blue. *P* value calculated using two-sided *t*-test. **c**, Scatter plot showing barcode-quantified tumorigenic potential and metastatic potential from subcutaneous and IC experiments respectively. **d**, Group1 of basal breast cancer pool (Extended Data Fig. 1a) was subjected to mammary fat pad injection, barcode quantitation through RNA-Seq, and cell number inference. Source data

To test the robustness of the MetMap dataset, we tested cell lines in two formats: in one, we injected all 498 cell lines as a single pool; in the other, we injected 5 pools of 25 lines, with each pool being injected into different mice (referred to as MetMap500 and MetMap125, respectively) (Fig. 2b). We similarly varied cell numbers, mouse age and cohort size to determine whether results varied substantially with these parameters. We observed strong correlation of the metastatic potential despite differences in experimental conditions (Fig. 2c), suggesting that the approach is extremely robust. We also note that intracardiac injection enabled the evaluation of many more cell lines in vivo compared with subcutaneous injection. Specifically, we recovered an average of 197 cell lines per mouse following intracardiac injection, whereas an average of 42 cell lines were recovered following subcutaneous injection (Extended Data Fig. 3a–c). We suspect that this difference is explained by the local competition for nutrients and other microenvironmental factors in the subcutaneous setting, whereas the spatial separation of tumour cells delivered through the intracardiac route minimizes such competition. A similarly reduced diversity was observed in the orthotopic setting, where injection of a pool of nine breast cancer cell lines into the mammary fat pad resulted in a single cell line dominating the resulting tumour (Extended Data Fig. 3d).

**Fig. 3:**
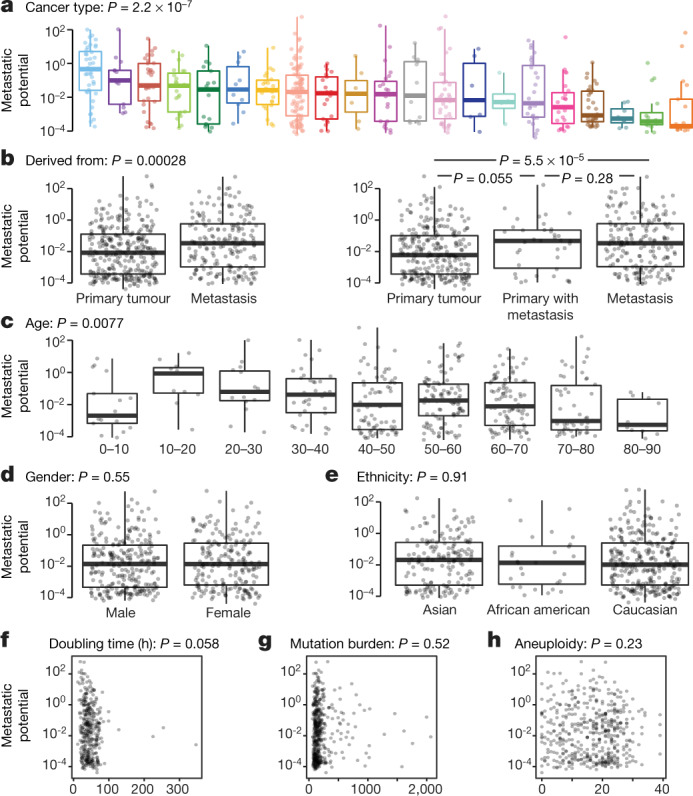
Clinical correlates of metastatic potential. **a**–**e**, Single-variate correlation of different clinical parameters with overall metastatic potential from MetMap500 data. Primary with metastasis indicates that the cell line was derived from the primary tumour and the donor developed metastasis at diagnosis or later. In box plots, boxes display quartiles of the data; outlier points extend beyond 1.5× interquartile ranges from either hinge. Cancer lineage is colour-coded as in Fig. 2a. **f**–**h**, Single-variate correlation of cell doubling, mutation burden and aneuploidy status with overall metastatic potential from MetMap500 data. **f**, Doubling time in hours. **g**, Mutation burden quantified by somatic mutations from exon-sequencing data. **h**, Aneuploidy quantified by chromosome-arm-level events from exon-sequencing data. Each dot represents a cell line. Source data.

**Extended Data Fig. 4:**
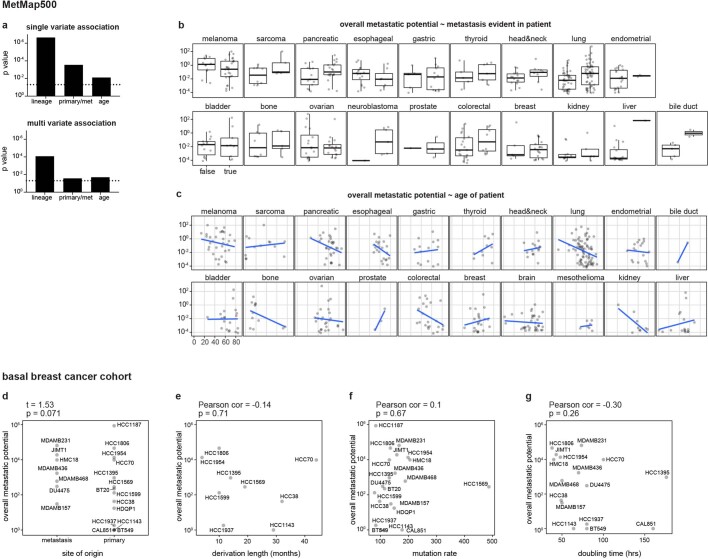
Association of overall metastatic potential with clinical parameters. **a**. Bar plots showing significance of single variate and multi variate association analysis with metastatic potential in MetMap500. *P* values are calculated using linear regression and Anova (type II) of the linear models. The dotted lines indicate 0.05 cutoff. **b**. Box plots showing metastatic potential of cell lines stratified by metastasis status in the corresponding patients and cancer lineage. Box plots display quartiles of the data. Outlier points extend beyond 1.5 × interquartile ranges from either hinge. **c**, Scatter plots showing correlation of metastatic potential with patient age, stratified by cancer lineage. An inverse correlation was observed in several cancer types. **d**–**g**, Correlation of overall metastatic potential with derived site (**d**), time length in culture to derive the cell lines (**e**), mutation burden (**f**) and cell doubling (**g**) in the 21 basal breast cancer cohort. **d**, *P* value calculated using two-sided *t*-test. **e**–**g**, Pearson’s correlation coefficients and test *P* values are presented. Source data

To determine whether the MetMap reflects the metastatic behaviour of human cancers, we analysed available clinical annotations of the cell lines (Fig. 3a–e, Extended Data Fig. 4). We found statistically significant associations with tumour lineage, the site from which the cell line was derived (primary tumour versus metastatic lesion) and patient age. There was no association between metastatic potential and gender or ethnicity. As expected, metastatic potential was higher in certain tumour types, such as melanoma and pancreatic cancer, which also tend to develop metastasis in the human disease setting^13^. By contrast, cell lines derived from brain tumours were generally non-metastatic, reflective of their tendency to not undergo haematogenous spread^14,15^. Similarly, the DU145 prostate cancer cell line, derived from a brain metastasis lesion^16^, exhibited brain metastasis in our experimental system. Cell lines derived from metastases showed higher metastatic potential than lines derived from primary tumours, although lines derived from primary tumours known to later give rise to metastases in patients were metastatic in the MetMap (Fig. 3b), consistent with previously reported suggestions that metastatic potential is already encoded in primary tumours^17–19^. The association between decreased metastatic potential and increased patient age was unexpected (Fig. 3c), and its basis remains to be determined.

Perhaps most importantly, extensive variation in metastatic potential was observed within individual lineages, making it possible to search for associations between metastasis propensity and genomic features of the tumours. Of note, metastatic potential was not simply explained by proliferation rate or mutational burden (Fig. 3f–h, Extended Data Fig. 4f, g), suggesting that more subtle molecular determinants of metastasis were involved.

## Molecular correlates of brain metastasis

To develop mechanistic insights, we focused on breast cancer and its potential for brain metastasis (Fig. 1b), because brain metastasis is a feature of some—but not all—breast cancers, and little is known about the underlying factors that could inform therapeutic approaches^20,21^. We therefore undertook a systematic and unbiased comparison of the molecular features that distinguished brain metastatic versus non-metastatic lines, using genomic data available for each of the cell lines.

**Fig. 4:**
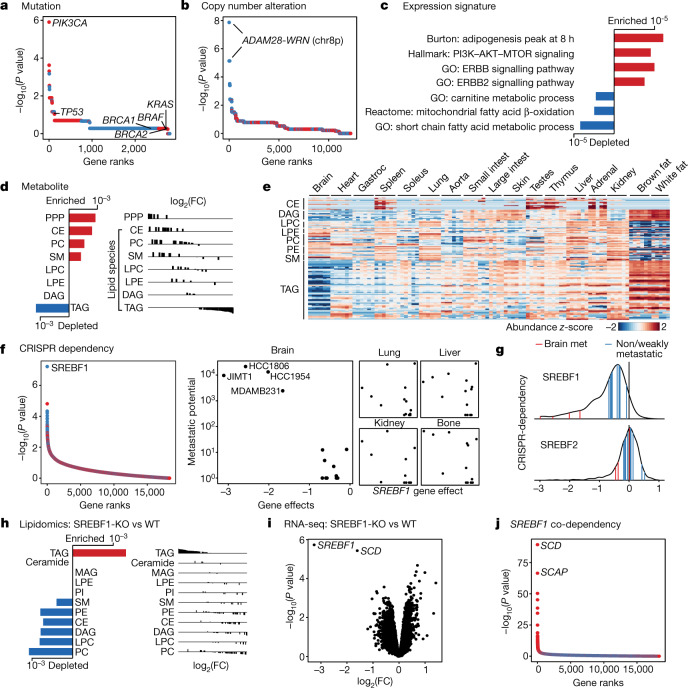
An altered lipid-metabolism state associates with brain metastatic potential in basal-like breast cancer. **a**, Somatic mutations that associate with brain metastatic potential in the basal-like breast cancer cohort. The top correlate, *PIK3CA*, reaches statistical significance (FDR = 0.0034, highlighted in bold). All *PIK3CA* mutations are activating. Positive correlations are in red, negative correlations are in blue. Selected known oncogenes or tumour suppressors in basal-like breast cancer are presented for comparison. **b**, Alterations in copy number that associate with brain metastatic potential. The top correlates cluster in chr 8p12–8p21.2 (FDR = 0.0017, highlighted in bold). **c**, Gene-expression signatures that associate with brain metastatic potential. Bars indicate *P* values. Expression signature scores were projected for each cell line with their in vitro RNA-seq data and used for regression analysis. GO (Gene Ontology), Hallmark, Reactome and Burton are gene sets in the MSigDB gene set enrichment analysis (GSEA) collection. **d**, Lipid-metabolite species that associate with brain metastatic potential. Bars indicate *P* values. Lipid metabolites measured by mass spectrometry were grouped by species, and enrichment analysis of the species was performed using GSEA. CE, cholesterol ester; PC, phosphatidylcholine; SM, sphingomyelin; LPC, lysophosphatidylcholine; LPE, lysophosphatidylethanolamine; DAG, diacylglycerol; PPP, pentose phosphate pathway metabolites. **e**, Heat map presenting distribution of lipid species measured by mass spectrometry from different mouse tissues. Gastroc, gastrocnemius. **f**, CRISPR gene dependencies that associate with brain metastatic potential. The top gene, *SREBF1* (FDR = 0.001), is a selective dependency in highly brain metastatic lines. Positive correlations are in red, negative correlations are in blue. **g**, Distribution of *SREBF1* (top) and *SREBF2* (bottom) dependencies across 688 human cancer cell lines. The positions of highly brain metastatic (met) breast lines are highlighted in red, whereas weakly metastatic or non-brain metastatic breast lines are highlighted in blue. **h**, Consensus alterations in lipid species abundance upon *SREBF1* knockout (KO) in JIMT1 and HCC1806, two brain metastatic cell lines. Bars indicate adjusted *P* values. Lipid metabolites measured by mass spectrometry were grouped by species, and enrichment analysis of the species was performed using GSEA. WT, wild type. **i**, Consensus gene-expression changes upon *SREBF1* knockout in JIMT1, HCC1806, HCC1954 and MDAMB231, four brain metastatic cell lines. The two top genes are *SREBF1* and *SCD* (FDR <0.05, highlighted in bold). **j**, Co-dependencies of *SREBF1* across 688 human cancer cell lines in genome-wide CRISPR viability screen. The two top genes are *SCD* and *SCAP* (FDR < 1 × 10^−60^, highlighted in bold). Source data.

**Extended Data Fig. 5:**
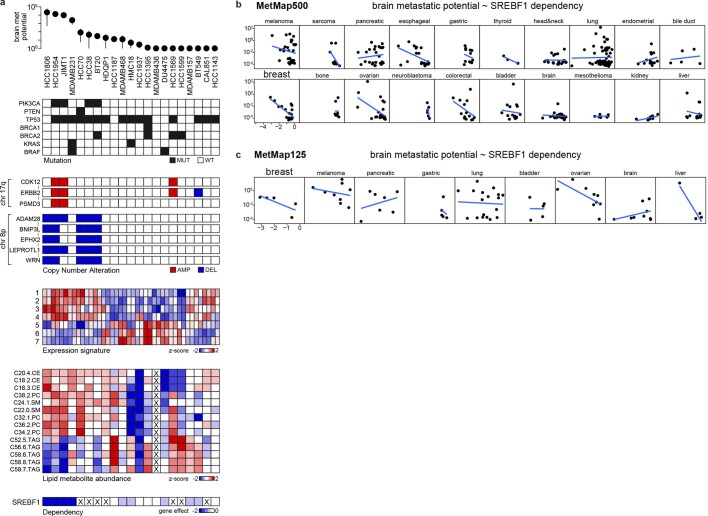
Genetic correlates of brain metastatic potential in basal-like breast cancer. **a**. A line-by-line view of brain metastatic potential and its associated features at genetic, expression, metabolite, and gene dependency levels. Mutation: mutant (MUT), wild-type (WT). Copy number: data are binarized, with deletion (DEL) cutoff < = -1 and amplification (AMP) cutoff > = 1. Expression signatures: 1. Hallmark: PI3K/AKT/MTOR signalling, 2. GO: ERBB signalling pathway, 3. GO: ERBB2 signalling pathway, 4. Burton: adipogenesis peak at 8hr, 5. GO: carnitine metabolic process, 6. Reactome: mitochondrial fatty acid beta oxidation, 7. GO: short chain fatty acid metabolic process. Data not available for the cell lines are marked with X. **b**, **c**, Scatter plots showing the correlation of SREBF1 in vitro dependency and brain metastatic potential in MetMap500 (a) and MetMap125 (b). Strong inverse correlation was observed for breast cancer in both datasets. Each dot represents a cell line. Source data

At the level of somatic mutations, *PIK3CA* was the top associated correlate: 4 out of 7 brain metastatic lines contained a *PIK3CA* mutation, compared with 0 out of 14 non-metastatic or weakly metastatic lines (false discovery rate (FDR) = 0.0034) (Fig. 4a, Extended Data Fig. 5a). A fifth line, HCC70, has a loss-of-function mutation in *PTEN*. PI3K is a principal downstream mediator of ERBB2 (also known as HER2), which itself has been reported to be associated with brain metastasis in humans^11,20^. Indeed, two of the brain metastatic cell lines (JIMT1 and HCC1954) also contain typical *ERBB2* gene amplifications (Extended Data Fig. 5a).

At the level of DNA copy number, we observed an association between metastatic potential and deletions of chromosome 8p12–8p21.2 (referred to as 8p) (FDR = 0.0017) (Fig. 4b). Five out of seven brain metastatic breast cancer cell lines contained deletions in this region, compared with 0 out of 14 non-metastatic lines (Extended Data Fig. 5a). A sixth metastatic line, JIMT1, has a small deletion within this commonly deleted region.

**Extended Data Fig. 6:**
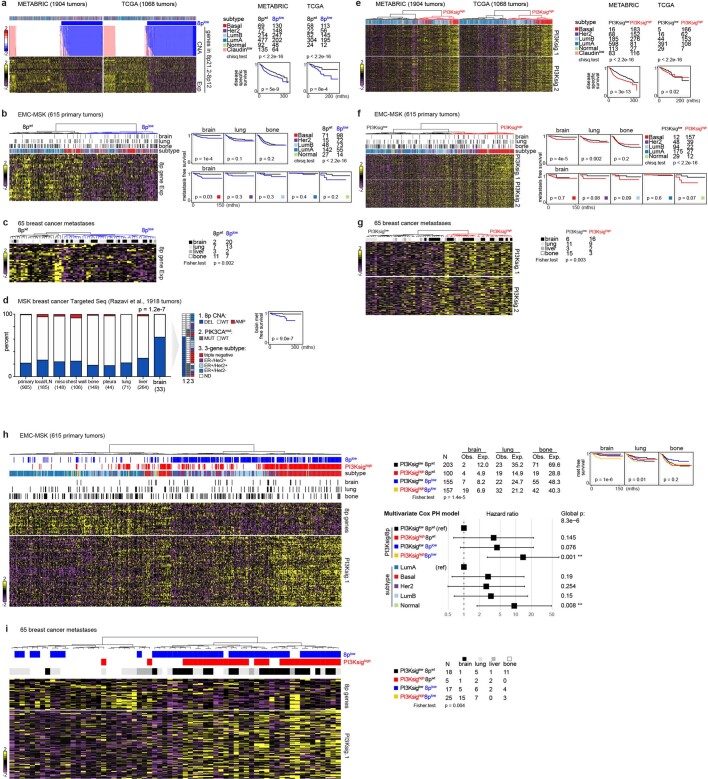
Association of chr 8p gene copy number status and PI3K-response signatures with brain metastasis in clinical breast cancer specimens. **a**, Heat maps showing coordinated expression of chr 8p genes mirrored their copy number status in the two large breast cancer datasets, METABRIC and TCGA. The 8p^low^ cluster is defined by CNA data. The right panel shows distribution of 8p^low^ cluster in different breast cancer subtypes and its association with disease specific survival. *P* values calculated using two-sided, log-rank tests. CNA, Copy Number Alteration. Exp, RNA-Seq Expression. **b**, Hierarchical clustering of primary breast tumours by 8p gene expression in the EMC-MSK dataset. The 8p^low^ cluster is enriched in tumours that developed brain metastasis, but not lung or bone metastasis. The right panel shows organ-specific metastasis free survival curves stratified by 8p^low^ status. The 8p^low^ cluster displays poorer brain metastasis compared to the 8p^WT^ cluster. Brain metastasis free survival curves stratified by 8p^low^ status in different subtypes is also presented. *P* values calculated using two-sided, log-rank tests. **c**, Hierarchical clustering of breast cancer metastases by 8p gene expression, with the 8p^low^ cluster being enriched in brain metastases. **d**, Chr 8p CNA status determined by Targeted Seq in the MSK metastatic breast cancer dataset. Brain metastases are enriched in chr 8p deletion compared to primary tumour, local recurrence and metastases at other sites. The 8p^low^ cluster predicts poor brain metastasis free survival. *P* values calculated using two-sided, log-rank tests. LN, lymph node. **e**, Heat maps showing co-regulated patterns of two independent PI3K-response signatures in METABRIC and TCGA breast cancer datasets. PI3Ksig.1 was generated by overexpression of PIK3CA^mut^ in breast epithelial cells. PI3Ksig.2 was generated by PI3K inhibitor treatment in the CMap database. The right panel shows distribution of PI3Ksig^high^ cluster in different breast cancer subtypes and its association with disease specific survival. *P* values calculated using two-sided, log-rank tests. **f**, Hierarchical clustering of primary breast tumours by PI3K signatures in the EMC-MSK dataset. The PI3Ksig^high^ cluster is enriched in tumours that developed brain metastasis. The right panel shows organ-specific metastasis free survival curves stratified by PI3K signatures. The PI3Ksig^high^ cluster displayed poorer brain metastasis. Brain metastasis free survival curves stratified by PI3K signatures in different subtypes is also presented. *P* values calculated using two-sided, log-rank tests. **g**, Hierarchical clustering of breast cancer metastases by PI3K signatures, with the PI3Ksig^high^ cluster being enriched in brain metastases. **h**, Heat maps showing significant yet non-complete overlap between 8p^low^ and PI3Ksig^high^ clusters in the EMC-MSK dataset. 8p^low^ and PI3Ksig^high^ clusters co-capture a subset of patients with the worst brain metastasis prognosis. *P* values calculated using two-sided, log-rank tests. The lower panel presents a Cox proportional-hazards model of brain metastasis free survival using multi variates – 8p, PI3Ksig, and breast cancer subtype. The 8p^low^/PI3Ksig^high^ cluster is the most associated with brain metastasis. i. 8p^low^ and PI3Ksig^high^ clusters co-capture the majority of brain metastasis samples. Source data

To ascertain the clinical relevance of these associations, we analysed clinical breast cancer datasets for which metastasis information was available^18^. We observed a strong correlation between 8p copy number and gene expression in the METABRIC and TCGA datasets^22,23^ (Extended Data Fig. 6a), thereby validating 8p expression as a surrogate for copy number in datasets for which copy number data were not available. Coordinated expression of 8p genes stratified tumours into two clusters, with the low-expressing cluster showing enrichment in brain metastasis and lower brain metastasis-free survival (Extended Data Fig. 6b). Whereas 8p loss was more frequent in basal-subtype breast cancer (known to have poor prognosis), 8p loss remained significantly associated with brain metastases within basal tumours. A similar trend was seen in other subtypes, but the sample size was too small to reach statistical significance. Concordant with these findings, the 8p-low signature was strongly enriched in brain metastasis lesions compared with extracranial metastases or primary tumours^24^ (Extended Data Fig. 6c, d). Similarly, we observed that response signatures^25,26^ indicating PI3K activation are associated with brain metastases (Extended Data Fig. 6e–g). The PI3K-high signature tended to co-occur with the 8p-low signature, and the overlapping events captured the majority of patients with brain metastases (Extended Data Fig. 6h, i). These results established the validity of the MetMap experimental system for discovery.

## Lipid metabolism and brain metastasis

Confirming these genetic findings, expression analysis revealed enrichment of PI3K and ERBB2 signatures in the brain metastatic cell lines (Fig. 4c). Furthermore, we observed a strong association between brain metastatic potential and a lipid-synthesis signature (Fig. 4c), which has been reported in association with both PI3K activation and 8p-deletion^27,28^. To investigate a potential role of lipid metabolism in breast-cancer brain metastatic potential, we analysed the abundance of lipid metabolites across the cell lines^29^. We observed increased levels of cholesterol species in highly brain metastatic cells (Fig. 4d). In addition to cholesterols, membrane lipids including phosphatidylcholine and sphingomyelin were similarly more abundant, as were metabolites associated with the pentose phosphate pathway^30^, which can support cholesterol and lipid synthesis. By contrast, we observed global decreases in levels of triacylglycerols (TAGs) in brain metastatic cells (Fig. 4d). Non-brain metastatic cells had higher levels of TAGs and contained a fatty acid oxidation signature (Fig. 4c). Metabolite profiling of normal mouse tissues^31^ showed that the brain has markedly lower levels of TAGs compared with other tissues (Fig. 4e). This reflects brain physiology, whereby instead of storing fatty acids as TAGs, the brain accumulates specialized lipids to support neural activity and brain function^32^. One possibility is that for breast cancer cells to survive in the brain microenvironment, where TAGs and other storage lipids present in other tissues are not abundant, they must access lipids via de novo synthesis or another route, in line with the seed-and-soil hypothesis^33^.

To further investigate the characteristics of breast cancer cell lines capable of brain metastasis, we analysed genome-wide CRISPR–Cas9 viability-screening data^34^ to identify gene vulnerabilities associated with the brain metastatic state. We identified *SREBF1* as the top-correlated dependency with brain metastasis (FDR = 0.001) (Fig. 4f). SREBF1 is a pivotal transcription factor that mediates lipid synthesis downstream of the PI3K pathway^27,35^. *SREBF1* was selectively required for growth of brain metastatic lines in culture compared with breast cancer lines with low or no brain metastatic potential. The association was specific to brain, as no association was observed between *SREBF1* essentiality and metastasis to other organs (Fig. 4f). This *SREBF1*–breast-cancer brain metastasis association was also recovered in the MetMap500 dataset, indicating strong reproducibility of the finding (Extended Data Fig. 5b, c). Of note, the *SREBF1* paralogue *SREBF2* showed no association between its essentiality in culture and metastatic potential (Fig. 4g).

**Extended Data Fig. 7:**
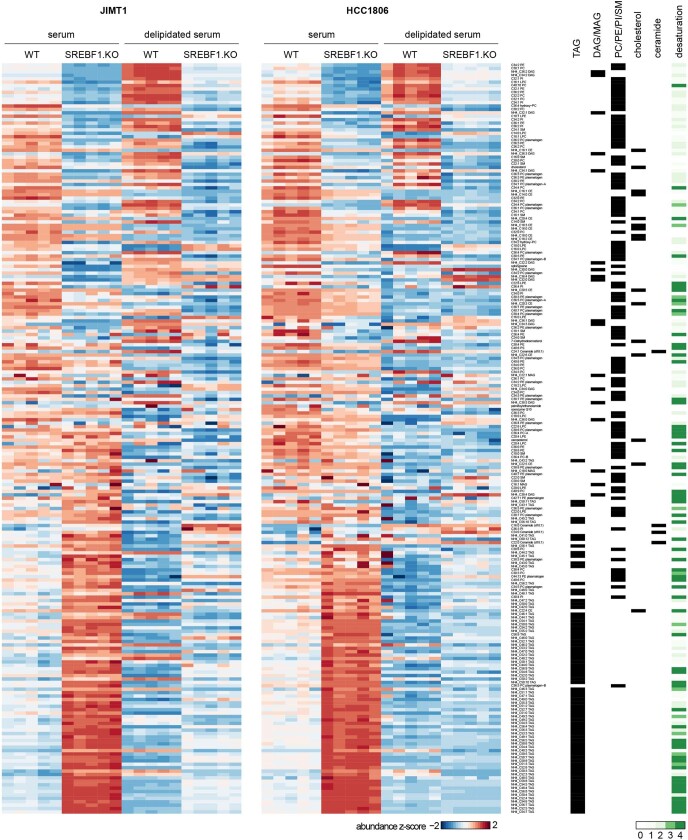
Lipid metabolite profile changes upon SREBF1 knockout. Heat maps showing relative lipid abundance in cells cultured in medium supplemented with serum or delipidated serum. SREBF1-WT and SREBF1-KO of JIMT1 (PIK3CA^mut^) and HCC1806 (8p^low^) were used. Lipid species groupings and lipid desaturation levels are also presented. WT, wild-type; KO, knockout. Source data

To investigate the role of *SREBF1* in affecting the lipid phenotype observed in brain metastatic cells, we performed lipidomics after knocking out *SREBF1* in JIMT1 and HCC1806 cells using CRISPR–Cas9. *SREBF1* knockout resulted in a marked shift in intracellular lipid content, including a decrease in levels of cholesterol, membrane lipids and diacylglycerols (Fig. 4h). *SREBF1* knockout also resulted in an increase in intracellular TAG levels, presumably by scavenging TAGs from the lipid-rich serum added to the culture medium. To test this hypothesis, we repeated the experiment in culture medium prepared with delipidated serum, which prevented the increase in TAGs observed in *SREBF1*-knockout cells (Extended Data Fig. 7).

**Fig. 5:**
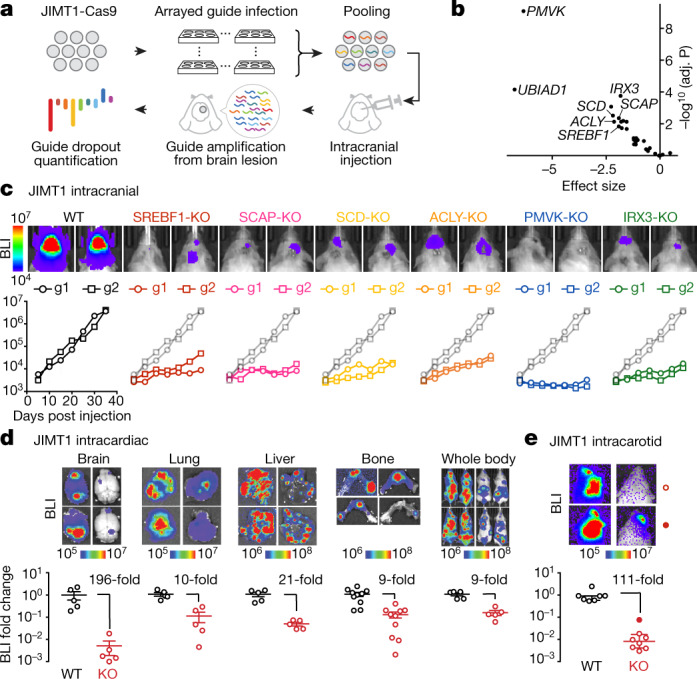
Investigation of lipid-metabolism genes in breast cancer brain metastasis. **a**, A schematic of an in vivo CRISPR screen investigating relative gene fitness in brain metastasis outgrowth. **b**, Volcano plot showing the result of a mini-pool in vivo CRISPR screen targeting 29 lipid-metabolism-related genes. Thirteen genes scored at FDR < 0.05, with selective hits highlighted. **c**, Individual gene validation of six hits by intracranial injection of JIMT1 edited cells. Cell outgrowth in brain metastasis was monitored by real-time BLI. Two independent guides per gene were tested, with one guide per-mouse. **d**, BLI and quantification of relative fold change in metastasis load in the organs of mice receiving intracardiac injection of wild-type (WT) or SREBF1-knockout (KO) JIMT1 cells. Data are mean ± s.e.m. Each group contains five mice. **e**, BLI and quantification of relative fold change in brain metastasis load in mice receiving intracarotid injection of wild-type or SREBF1-KO JIMT1 cells. Data are mean ± s.e.m. *n* = 7 (wild-type) and *n* = 8 (knockout) mice. Source data.

**Extended Data Fig. 8:**
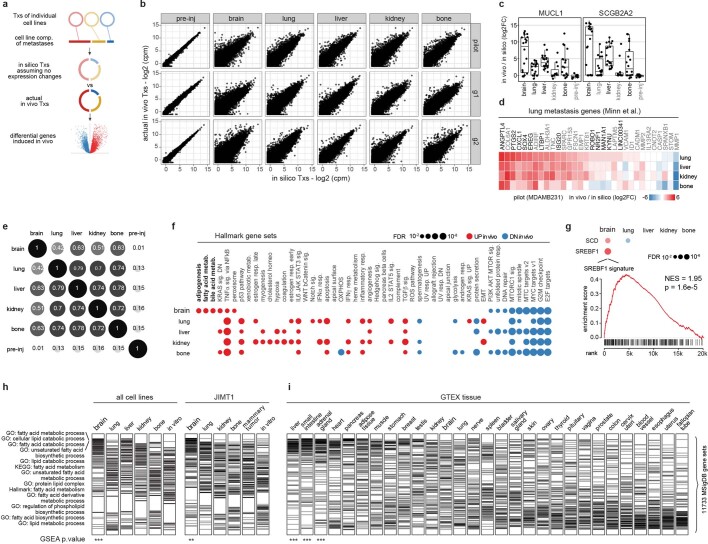
Analysis of multiplexed breast cancer metastasis in vivo transcriptomes. **a**, A schematic of the differential analysis approach for in vivo transcriptomes with mixtured cancer cell lines. An in silico transcriptome was modelled based on single cell line in vitro transcriptomes and cell line composition (comp.) of the metastasis sample. The in silico profile was then compared with the actual in vivo data in a paired-wise manner. **b**, Comparison of in silico modelled profiles to the actual pre-injected or in vivo metastasis sample profiles. The pre-injected populations are direct mixtures of in vitro cell lines and show tight correlation with in silico data. In vivo samples show large fold changes. **c**, Box plots showing log_2_ fold changes of MUCL1 and SCGB2A2 in in vivo metastasis samples and pre-injected cells. Each point represents a sample. Box plots display quartiles of the data. Outlier points extend beyond 1.5 × interquartile ranges from either hinges. **d**, Heat map showing log_2_ fold change of lung metastasis genes (Minn et al.) in lung, liver, kidney and bone metastasis samples from the pilot study, where MDAMB231 dominated the population. **e**, Correlation of gene expression changes in different metastasis sites. Pre-injected population had no expression change and thus showed no correlation with in vivo samples. Brain metastases showed weaker correlations with extracranial metastases. **f**, Bubble plot showing enrichment of Hallmark gene pathways (MSigDB) comparing in vivo expression of metastases at different organ sites to their in vitro counterparts. **g**, Bubble plot showing in vivo upregulation of SREBF1, SCD and SREBF1-response signature in brain metastases. **h**, **i**, GSEA analysis of lipid metabolism gene sets using in vivo RNA-Seq profiles combined by metastasis organ sites irrespective of sample or cell line composition (**h**). Gene sets related to lipid metabolism are selectively enriched on top in the brain but not in other organs or in vitro. Restricting analysis to JIMT1-dominant samples revealed a similar result. No enrichment was seen in normal brain when analysis was performed on GTEX normal tissue (**i**). Each tick represents a lipid metabolism gene set from MSigDB. ***, *P* = 0.001; ** = 0.01. Source data

**Extended Data Fig. 9:**
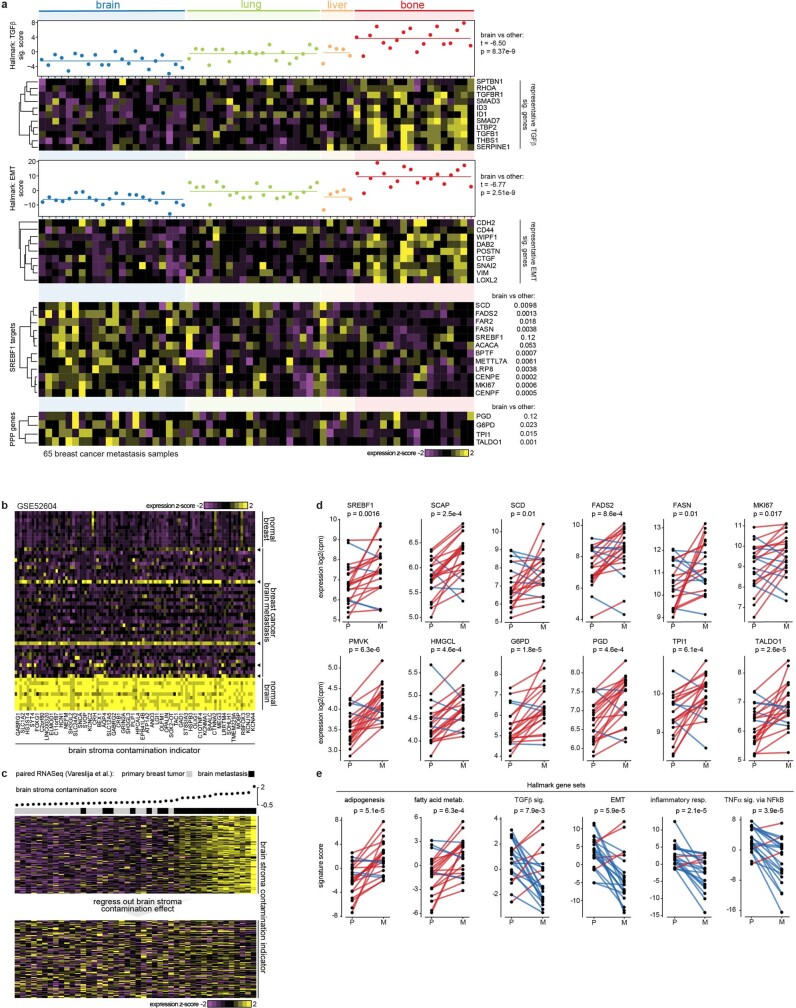
Expression of TGFβ signalling, EMT status, inflammatory response and lipid metabolism genes in clinical breast cancer metastasis specimens. **a**, Comparison of brain metastasis versus extracranial metastasis clinical samples. Lower expression of TGFβ signature genes and EMT signature genes in brain metastases than other metastasis sites. Enriched expression of selective SREBF1 target genes (including FASN, SCD, SREBF1 itself) and Pentose Phosphate Pathway (PPP) genes in brain metastases. **b**, **c**, A strategy to remove brain stroma contamination effect from brain metastasis expression profiles when performing comparison of paired primary breast tumour and brain metastasis clinical specimens. A gene signature indicating brain stroma contamination was derived from comparison of brain with breast and breast cancer brain metastasis (**b**). Arrowheads indicate a few brain metastasis samples with noticeable brain stroma contamination. A brain contamination score was calculated and its effect was regressed out in the RNASeq data of matched primary tumours and brain metastases (**c**). The heat map shows expression of brain stroma indicator before and after removal of the contamination effect. **d**, **e**, Paired comparison of primary breast tumour and brain metastasis clinical specimens after removal of brain stroma contamination. **d**, Lipid metabolism genes and PPP genes. **e**, Signature scores were projected for each sample using the corrected RNA-Seq profiles. P, Primary breast tumour; M, brain Metastasis; upregulation in red, downregulation in blue. *P* values calcutated using paired, two-sided *t*-tests. Source data

To further explore the role of SREBF1, we performed RNA-seq following *SREBF1* knockout and found *SCD*^35^ to be the most consistently downregulated gene (Fig. 4i). Consistent with this, *SCD* was the top co-dependency of *SREBF1* across 688 cell lines in the genome-wide CRISPR–Cas9 viability screens (Fig. 4j). The next highest scoring *SREBF1* co-dependency was *SCAP*, which encodes the upstream activator of *SREBF1*^35^. Comparison of gene expression in breast cancer cells grown in vitro or in the brain similarly showed that in the brain, cells adopted gene-expression signatures of adipogenesis, fatty acid metabolism and xenobiotic metabolism (Extended Data Fig. 8, Supplementary Note 3). The enrichment of lipid-metabolism signatures (including upregulation of *SREBF1* and *SCD*) was unique to brain compared with other sites of metastasis. Similar upregulation was also observed in brain metastases from patients compared with extracranial metastases or their matched primary tumours^36^ (Extended Data Fig. 9). Furthermore, the requirement for *SREBF1*, *SCD*, *SCAP* and other members of the lipid-metabolism pathway for brain metastasis formation was confirmed in both mini-pool and individual gene-knockout experiments (Fig. 5a–c, Supplementary Note 4). Together, these genetic, metabolic, transcriptomic and functional genomic evidence all point to an association between SREBF1-mediated lipid metabolism and brain metastasis.

**Extended Data Fig. 10:**
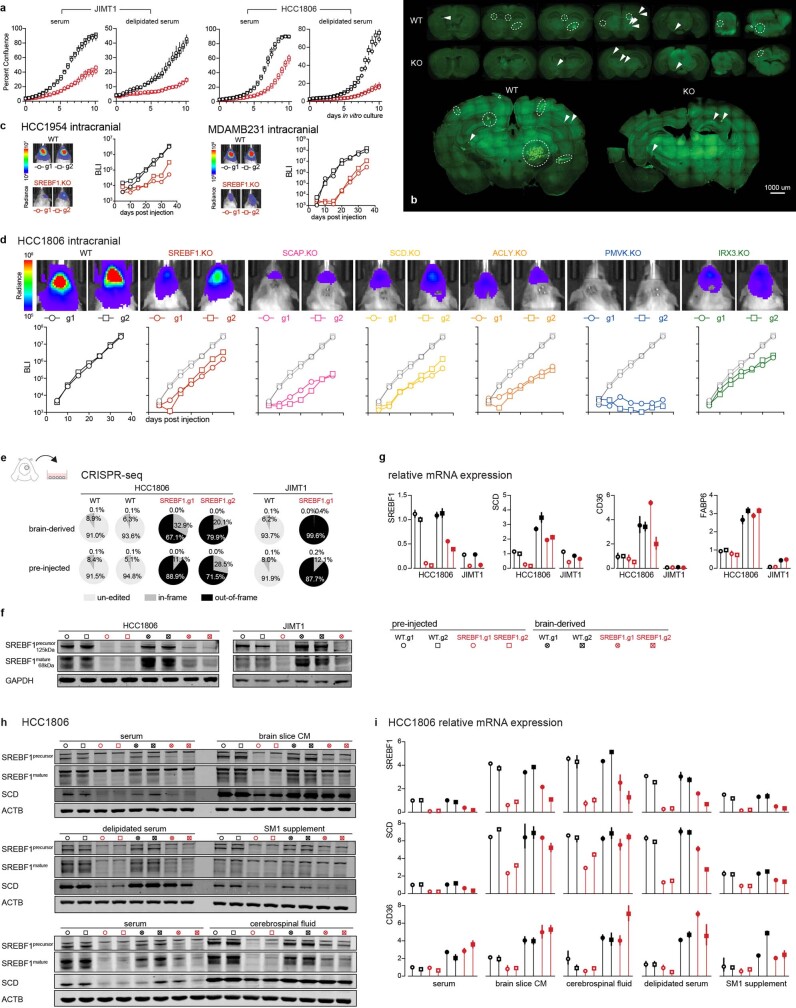
In vivo and in vitro effects of SREBF1 knockout. **a**, Growth kinetics of SREBF1-WT and -KO cells in in vitro culture medium with 10% serum or 10% delipidated serum. Cell growth was monitored by Incucyte real-time imaging. WT, wild-type, in black; KO, knockout, in red. Two independent guides were used per group. **b**, Fluorescence imaging of metastases in serial brain sections from mice receiving intracardiac injection of JIMT1 SREBF1-WT or -KO cells (Fig. 5d). Confocal tile scans of representative sections are presented at the lower panel. GFP+ signals indicate cancer lesions. Circles highlight macro-metastatic lesions and arrows indicate micro lesions. **c**, **d**, One-by-one assessment of lipid metabolism gene fitness in additional brain metastatic cell lines through intracranial injection. SREBF1 was tested for HCC1954, MDAMB231 (**c**) and HCC1806. Additional genes were tested for HCC1806 (**d**). Cell outgrowth in brain metastasis was monitored by real-time BLI. Two independent guides per gene were tested, in a one guide one mouse fashion. **e**–**g**, Outgrowing (HCC1806) or residual (JIMT1) SREBF1-KO cells from brain metastases were derived for CRISPR-seq (**e**), western blot (**f**), and RT–qPCR (**g**) assays. **e**, CRISPR-seq quantifying SREBF1 gene editing efficiencies of brain-derived and pre-injected cells. **f**, Western blot quantifying SREBF1 protein levels. **g**, RT–qPCR quantifying relative expression of SREBF1, SCD, CD36, FABP6 in brain-derived versus pre-injected cells. Pre-injected WT HCC1806 was used as reference. **h**, **i**, Brain-derived and pre-injected HCC1806 cells were cultured in brain-slice-conditioned medium (CM) or medium supplemented with cerebrospinal fluid, or serum, or delipidated serum, or SM1 supplement, and western blot (**h**) or RT–qPCR was performed (**i**). SREBF1, SCD and CD36 were upregulated when cells were cultured in brain slice CM, cerebrospinal fluid, and delipidated serum. Brain-derived SREBF1-KO cells were better at inducing SCD and CD36, in comparison to pre-injected SREBF1-KO cells. Experiments were performed twice independently with similar results. Source data

Given the observation that *SREBF1* knockout resulted in a viability defect in vitro (Extended Data Fig. 10a), we compared the relative effect of knockout on metastasis to different organs, to determine whether the viability defect was preferentially observed in brain (Fig. 5d). Five weeks following intracardiac injection of *SREBF1*-knockout cells, we observed a marked defect in brain metastasis (196-fold reduction), compared with a modest defect in other organs (9–21 fold) (Fig. 5d). Histologic examination of brains from xenografted mice revealed large metastatic lesions in mice receiving wild-type cells, whereas those receiving *SREBF1*-knockout cells contained micrometastases (Extended Data Fig. 10b), suggesting that *SREBF1* is not required for seeding the brain, but rather for proliferation in the brain microenvironment. Consistent with this hypothesis, injection of tumour cells into the carotid artery increased the probability of seeding the brain, but nevertheless a marked growth defect was still observed in *SREBF1*-knockout cells (Fig. 5e).

To determine the generality of the *SREBF1* requirement for breast cancer growth in the brain, we knocked out *SREBF1* in additional brain metastatic lines including HCC1954, MDAMB231 and HCC1806 using CRISPR–Cas9. As with JIMT1, a significant inhibition in brain metastatic growth was also observed in these lines, although the magnitude and duration of growth inhibition varied (Extended Data Fig. 10c, d). The least responsive cell line was HCC1806, in which *SREBF1*-knockout cells displayed a brain growth defect for the first week, but then assumed a growth trajectory that paralleled wild-type cells. This restoration of growth was not explained by reversion of the genome editing, as brain metastases at the end of the experiment showed evidence of editing at the *SREBF1* locus and minimal *SREBF1* protein expression (Extended Data Fig. 10e, f). Instead, we found that the *SREBF1*-independent growth was associated with upregulation of the fatty acid transporter CD36 and the fatty acid-binding protein FABP6 (Extended Data Fig. 10g). Of note, culture of HCC1806 in mouse brain-slice-conditioned medium similarly resulted in upregulation of SCD and CD36 expression (Extended Data Fig. 10h, i). JIMT1 cells did not upregulate CD36 or FABP6 expression following *SREBF1* knockout (Extended Data Fig. 10g), perhaps explaining their inability to survive in the brain. Together, these results further demonstrate the relationship between lipid metabolism and brain metastasis, as cells under the selective pressure of *SREBF1* loss must acquire lipids by other means to survive in the brain microenvironment.

## Discussion

This work describes MetMap as an approach for large-scale in vivo characterization of human cancer cell lines. The MetMap resource (available at https://pubs.broadinstitute.org/metmap) currently includes metastasis profiles of 500 cell lines spanning 21 tumour types, providing a large repertoire of models for exploration of metastasis mechanisms. A limitation of the use of human cell lines for such experiments is that they require the use of immunodeficient mice. The extent to which the immune system has a role in mediating patterns of metastasis remains to be determined^37^.

We followed up only a small proportion of the MetMap findings—specifically, breast cancer metastasis to brain. Multiple lines of experimental and clinical evidence pointed to a role of lipid metabolism in governing the ability of cells to survive in the brain microenvironment. The importance of lipid metabolism in cancer has been highlighted by a number of studies, but its role in brain metastasis has, to our knowledge, not been fully appreciated^38–41^. The possibility that interfering with lipid or cholesterol metabolism might abrogate metastatic growth in the brain is particularly intriguing. More generally, this work illustrates the complex interplay between cancer cell growth and the tissue microenvironment.

## Methods

No statistical methods were used to predetermine sample size. The experiments were not randomized. The investigators were not blinded to allocation during experiments and outcome assessment.

### Breast cancer cell lines and barcoding

Breast cell lines were cultured under the recommended conditions from CCLE (https://portals.broadinstitute.org/ccle). Cell line identities were confirmed by SNP fingerprinting as well as RNA-seq, and compared to the CCLE results. All cell lines were tested negative for mycoplasma. The fluorescence-luciferase-barcode (FLB) construct was engineered using the FUW lentiviral vector backbone (a gift from D. Baltimore; Addgene plasmid no. 14882). Barcodes 26 nucleotides in length were designed using barcode_generator.py (v.2.8; http://comailab.genomecenter.ucdavis.edu/index.php/), and cloned into the landing pad C-terminal to the TGA stop codon of fluorescence luciferase using Gibson assembly (New England Biolabs). Lentivirus preparation and cell infection were performed according to published protocols available at http://www.broadinstitute.org/rnai. Infected cells were analysed by FACS with a fixed gate for GFP or mCherry, using a Sony SH4800 sorter.

### Animal studies

Animal work was performed in accordance with a protocol approved by the Broad Institute Institutional Animal Care and Use Committee (IACUC). NSG female mice (The Jackson Laboratory) at 5–6 weeks of age were used. Cancer cells were suspended in PBS, 0.4% BSA and 100 μl of cell suspensions were injected into the left ventricle of anaesthetized mice (ketamine 100 mg kg^−1^; xylazine 10 mg kg^−1^). In vivo metastasis progression was monitored via real-time BLI using the IVIS SpectrumCT Imaging System (PerkinElmer) on a weekly basis. Mice were anaesthetized with inhaling isoflurane, injected intraperitoneally with D-Luciferin (150 mg kg^−1^), and imaged with the auto exposure setting in prone and supine positions. At the end point, ex vivo BLI was performed by submerging the excised organs in DMEM/F12 medium (Thermo Fisher Scientific) containing D-Luciferin for 10 min and imaged with the auto exposure setting. BLI analysis was performed using Living Image software (v.4.5, PerkinElmer). In the case of breast cancer cohort study (pilot, group 1 and group 2 in Fig. 1, Extended Data Fig. 1), cell lines were mixed at an equal ratio immediately before animal injection, and cell line pools containing 2 × 10^4^ cells per barcoded line were injected. In the case of single breast cell line validation (Extended Data Fig. 1n), cell lines were injected individually at a density of 2 × 10^4^ cells, to be comparable with the pooled experiments. In the case of MetMap125 (Fig. 2, Extended Data Fig. 2), PRISM pools of 25 cell lines were used, and 2.5 × 10^5^ total cells were injected per mouse, corresponding to 1 × 10^4^ cells per barcoded line. Five PRISM pools were injected separately into cohorts of 5–6-week-old NSG mice. In the case of MetMap500, 20 PRISM pools of 25 cell lines were combined to form a large pool of 498 cell lines. The large pool was injected into a cohort of 8–10-week-old NSG mice, with 2.5 × 10^5^ cells per mouse, equivalent to a density of 500 cells per line. Mammary fat pad and subcutaneous injections were performed with Matrigel (Corning) support, at a matching density to their intracardiac assays, respectively (Extended Data Fig. 3). For all pooled cell line experiments, mice were euthanized 5 weeks after injection, in a time-matched manner, unless they displayed severe paralysis or poor body condition, in which case they were euthanized earlier. Intracartoid injection of JIMT1 was performed following a published protocol^42^, at a density of 1 × 10^5^ cells per mouse, similar to the intracardiac injection (Fig. 5e). Intracranial injection was performed as previously described^43^, at a density of 1 × 10^3^ cells per perturbation per animal (Fig. 5a–c, Extended Data Fig. 10c, d).

### Tissue processing and cancer cell isolation from organs

Organs including brain, lung, liver, kidney were dissociated using gentleMACS Octo Dissociator with Heaters (Miltenyi Biotec). The optimized dissociation solutions and programs (Miltenyi Biotec) are listed in Supplementary Table 9. Bones (from both hind limbs) were chopped into fine pieces and incubated in the dissociation buffer with vigorous shaking. The dissociated cell suspensions were filtered using 100-μm filters, and washed with DMEM/F12 twice. Cell suspensions were then washed with staining buffer (PBS, 2 mM EDTA, 0.5% BSA), and incubated with mouse cell depletion beads according to the instructions (Miltenyi Biotec). Cell suspensions were subjected to negative selection using autoMACS Pro Separator (Miltenyi Biotec) to deplete mouse stroma. Brains were subjected to an additional myelin-debris-depletion step using myelin removal beads II (Miltenyi Biotec). The resultant cell suspensions were then analysed by FACS using a Sony SH4800 sorter, with the fixed gate for GFP or mCherry. DAPI staining was used to exclude dead cells. For bulk RNA-seq, cells were sorted to a single tube in PBS, 0.4% BSA and RNasin Plus RNase Inhibitor (Promega), centrifuged at 1,500 rpm for 10 min, and cell pellets were frozen at −80 °C for downstream use. For single-cell RNA-seq, single cells were sorted into 96-well plates containing cold TCL buffer (Qiagen) containing 1% β-mercaptoethanol, snap frozen on dry ice, and then stored at −80 °C. Ninety single cells were sorted per plate, the rest wells on the plate were used for negative and positive controls.

### RNA extraction, library preparation and sequencing

Individual cell lines, cell line pools before injection, and cells isolated from metastases were analysed by RNA-seq. RNA extraction was performed using Quick-RNA MicroPrep according to the manufacturer’s instructions (Zymo Research). RNA was quantified using an RNA 6000 Pico Kit on a 2100 Bioanalyzer (Agilent). RNA samples from cell numbers lower than 500 were not measured but all were used as input for library preparation. cDNA was synthesized using Clontech SmartSeq v.4 reagents from up to 2 ng RNA input according to the manufacturer’s instructions (Clontech). Full-length cDNA was fragmented to a mean size of 150 bp with a Covaris M220 ultrasonicator and Illumina libraries were prepared from 2 ng of sheared cDNA using Rubicon Genomics Thruplex DNaseq reagents according to the manufacturer’s protocol. The finished double-stranded DNA (dsDNA) libraries were quantified by Qubit fluorometer, Agilent TapeStation 2200, and RT–qPCR using the Kapa Biosystems library-quantification kit. Uniquely indexed libraries were pooled in equimolar ratios and sequenced on Illumina NextSeq500 runs with paired-end 75-bp reads at the Dana-Farber Cancer Institute Molecular Biology Core Facilities. RT–qPCR quantification of barcodes was performed using Maxima First Strand cDNA Synthesis Kit, Taqman Fast Advanced Master Mix, custom synthesized Taqman probes, and QuantStudio 6 PCR System (ThermoFisher Scientific). Single-cell RNA-seq was performed as previously described^44^.

### Bioinformatic analysis

#### Barcode quantification from RNA-Seq of metastases

Because the RNA-seq library preparation sheared the cDNA randomly into small pieces, demultiplexed RNA-seq reads were mapped to the barcode references using Bowtie 2 local mode^45^ for barcode detection and quantification. Mapped reads were filtered with the criteria that reads (either 5′ or 3′) must cover over 50% of the barcodes from either end, and counted using samtools. Barcode percentage corresponding to cell composition was calculated for single cell lines, pre-injected cell mixtures and in vivo metastasis samples.

#### Metastatic potential quantification and feature associations

For breast cohort study, metastatic potential of cell line *j* targeting organ *i, M*_*i,j*_ was calculated as: ${M}_{i,j}=\frac{1}{n}\mathop{\sum }\limits_{k=1}^{n}{c}_{i}{p}_{j}$, in which *c*_*i*_ is the total cancer cell number isolated from organ *i*, *p*_*j*_ is the fractional proportion of cell line *j* estimated by barcode quantification, and *n* is the number of replicates of mice. To identify features that associate with brain metastatic potential, a two-class comparison method was used^46^. The analysis was performed on mutation, copy number, expression, metabolite, and CRISPR-gene dependency (available at https://depmap.org/portal/). Copy number data were binarized using a cutoff of ≤−1 (loss) and ≥1 (gain).

#### Cancer transcriptomic analysis from RNA-seq of metastases

Potential mouse contaminating reads were removed by competitive mapping to the human/mouse hybrid genome using BBSplit (https://sourceforge.net/projects/bbmap/). Reads that uniquely mapped to the human genome were then used as input for mapping and gene-level counting with the RSEM package^47^. Gene count estimates were normalized using the TMM method in edgeR^48^. For differential analysis, to properly account for the cancer cell composition differences in each in vivo sample, an in silico modelled in vitro mixture was generated first. For each in silico metastasis model, the estimated expression *ĝ* of gene *i* is computed as a weighted average of the cell lines present in the corresponding in vivo sample: ${\hat{g}}_{i}=\mathop{\sum }\limits_{j=1}^{M}{g}_{i,j}{p}_{j},\,$in which *g*_*i,j*_ is the baseline in vitro expression of gene *i* in cell line *j* and *p*_*j*_ is the fractional proportion of cell line *j* in the in vivo sample, as estimated by barcode quantification, and *M* is the number of cell lines present in the in vivo sample. The in vivo and in silico counterpart were then compared using a paired design for each organ in voom-limma^46^. GSEA was performed using camera or GSEA-preranked method implemented in fgsea^46,49^. Single sample GSEA signature projection was performed using gsva package^50^. Gene-signature datasets were from MSigDB (https://www.gsea-msigdb.org/).

### PRISM in vivo assay

#### PRISM pool preparation

PRISM cell lines (source of each available at https://depmap.org/portal/) were adapted to the same culture conditions in phenol-red-free RPMI1640 medium (ThermoFisher Scientific) and barcoded as previously described^12^. SNP fingerprinting authentication was performed before and after barcoding. Mycoplasma contamination was examined (MycoScope, Genlantis) and only negative lines were used for experiments. These included eight oestrogen-receptor-positive breast cancer cell lines. Despite the lack of phenol red (a weak oestrogen) these breast cell lines maintained ESR1 positivity and expression of a downstream marker of its activity, FOXA1. This is probably explained by the remaining oestrogens in the fetal bovine serum (FBS). PRISM cell lines were pooled on the basis of their in vitro doubling bins, at equal number, in the format of 25 lines per pool, and cryopreserved until use. Cells were thawed and recovered for 48 h before in vivo injection. To form the large pool of 498 cell lines, 20 PRISM pools were mixed at equal total number immediately before injection.

#### Tissue processing, library preparation and sequencing

After in vivo experiments, organs were subjected to tissue dissociation, mouse stroma depletion, and the dissociated cell pellets were frozen at −80 °C as described above. The pellets (≤50 mg dry weight) were lysed in 200 μl freshly prepared lysis buffer with proteinase K, heat digested at 60 °C, and denatured at 95 °C for 10 min. Twenty microlitres of the lysates was used for barcode amplification per 100 μl PCR volume (multiple technical replicates per sample). PCR was performed using the following conditions: 95 °C for 3 min; 98 °C for 20 s, 57 °C for 15 s, 72 °C for 10 s (30 cycles); 72 °C for 5 min; 4 °C stop. PCR libraries were pooled, purified using Select-a-Size DNA Clean & Concentrator Kit (Zymo Research), and quantified using Qubit dsDNA HS Assay Kit (ThermoFisher Scientific) and a 2100 Bioanalyzer (Agilent). The purified 2 nM of libraries with 20% spike-in PhiX DNA were sequenced on Illumina MiSeq or HiSeq at 800 K mm^−2^ cluster density.

#### Metastatic potential quantification

Demultiplexed sequencing reads were mapped to the barcode reference to generate a table of cell line barcode counts for each sample/condition. Sequencing-depth normalized read counts were used for calculation of relative metastatic potential. Relative metastatic potential of cell line *j* targeting organ *i*, *rM*_*i,j*_, was defined as: $r{M}_{i,j}=\frac{1}{n}\mathop{\sum }\limits_{k=1}^{n}{c}_{i,j}/\frac{1}{m}\mathop{\sum }\limits_{k=1}^{m}{p}_{j}$, in which *c*_*i,j*_ is the read counts of cell line *j* from organ *i*, *p*_*j*_ is the read counts of cell line *j* from pre-injected population, *n* is the number of replicate samples of mice, *m* is the number of replicates of pre-injected population. Confidence intervals were calculated using bootstrap resampling.

### In vivo CRISPR screen and gene validation

CRISPR–Cas9 versions of cell lines were generated by infecting luciferized cells with Cas9-Blast lentivirus and selecting in 5 μg ml^−1^ blasticidin for 10 days with continuous passaging until non-infected controls were killed. For pooled in vivo screen, JIMT1–Cas9 cells were infected with a CRISPR guide library (Supplementary Table 10) in an arrayed-fashion in 6-well plates, and selected in 2 μg ml^−1^ puromycin for 4 days. At this time, non-infected controls were killed, and no growth defect was observed in the perturbed cell lines. Post antibiotic selection, cells were pooled and subjected to intracranial injection at 6 × 10^4^ cells per mouse in 1 μl PBS. This was equivalent to 1 × 10^3^ cells per guide on average per mouse. Intracranial growth was allowed to progress for 4 weeks, and brain tissues were processed adopting the workflow of PRISM in vivo assay, except that guides were amplified using primers targeting the guide vector. Demultiplexed sequencing reads were mapped to the guide reference to generate a table of barcode counts for each guide for each sample. Sequencing-depth was normalized using the upper-quartile method and relative depletion was quantified using a linear model in limma^46^. For individual gene validation (Fig. 5c, Extended Data Fig. 10c, d), Cas9-expressing cells of different cell lines were infected with corresponding guides, selected in 2 μg ml^−1^ puromycin for 4 days, and subjected to intracranial injection at 1 × 10^3^ cells per mouse in 1 μl PBS. Two independent guides per gene were tested, with one mouse per guide. Intracranial growth was monitored by BLI following injection.

### Liquid chromatography–mass spectrometry lipidomics

Positive ion mode analyses of polar and nonpolar lipids (C8-pos) were conducted using a liquid chromatography–mass spectrometry (LC–MS) system composed of a Shimadzu Nexera X2 U-HPLC (Shimadzu) coupled to an Exactive Plus orbitrap mass spectrometer (ThermoFisher Scientific). Cellular extracts were collected from 6-well plate culture, in LC-MS-grade isopropanol (Sigma-Aldrich) containing an internal standard 1,2-didodecanoyl-*sn*-glycero-3-phosphocholine (Avanti Polar Lipids). Extracts were centrifuged for 10 min at 10,000*g* to remove residual cellular debris. After centrifugation, supernatants were injected directly onto a 100 × 2.1 mm, 1.7-μm ACQUITY BEH C8 column (Waters). The column was eluted isocratically with 80% mobile phase A (95:5:0.1 v/v/v 10 mM ammonium acetate/methanol/formic acid) for 1 min followed by a linear gradient to 80% mobile phase B (99.9:0.1 v/v methanol/formic acid) over 2 min, a linear gradient to 100% mobile phase B over 7 min, then 3 min at 100% mobile phase B. Mass spectrometry analyses were performed using electrospray ionization in the positive ion mode using full scan analysis over 200 to 1,000 *m*/*z* at 70,000 resolution and 3 Hz data acquisition rate. Other mass spectrometry settings were as follows: sheath gas 50, in source collision-induced dissociation 5 eV, sweep gas 5, spray voltage 3 kV, capillary temperature 300 °C, S-lens RF 60, heater temperature 300 °C, microscans 1, automatic gain control target 10^6^, and maximum ion time 100 ms. Lipid identities were determined on the basis of comparison to reference standards and reference plasma extracts and were denoted by the total number of carbons in the lipid acyl chain(s) and total number of double bonds in the lipid acyl chain(s).

### Western blot

Protein lysates were prepared in RIPA lysis buffer (ThermoFisher Scientific) with cOmplete Mini EDTA-free Protease Inhibitor Cocktail (Roche). Western blot was performed using NuPAGE gel (ThermoFisher Scientific) with wet tank blotting (Bio-Rad) and Odyssey detection system (LI-COR). SREBF1 primary antibody (14088-1-AP, Proteintech), SCD (CD.E10) antibody (ab19862, Abcam), GAPDH (D16H11) XP rabbit monoclonal antibody (5174S, Cell Signaling), β-actin (8H10D10) mouse monoclonal antibody (3700S, Cell Signaling), and IRDye 800CW goat anti-mouse IgG (926-32210, LI-COR), IRDye 680RD goat anti-rabbit IgG (926-68071, LI-COR) secondary antibodies were used. Western blot was performed for cells cultured in different medium conditions. These include RPMI1640 with 10% FBS, with 10% delipidated FBS, with 10% human cerebrospinal fluid (991-19-P-5, Lee BioSolutions), or with 1% SM1 supplement (05711, STEMCELL Tech), or brain-slice-conditioned medium. Brain-slice-conditioned medium was prepared by submerging brain slices (150 μm) in RPMI1640 (no serum) for 48 h. Delipidated FBS was prepared as described^51^.

### Clinical data analysis

METABRIC, TCGA and MSK targeted-sequencing breast cancer datasets were downloaded from cBioPortal^52^. The EMC-MSK dataset including 615 primary tumours (GSE2035, GSE2603, GSE5327 and GSE12276) and the 65-metastasis-sample dataset (GSE14020) were collected and processed as previously described^18^. Paired primary breast tumour and brain metastasis RNA-seq was obtained from ref. ^36^. To exclude the confounding effect of brain stroma contamination in this dataset, a contamination indicator generated from GSE52604 was applied, and the contaminating effect was regressed out, generating a corrected gene matrix. PI3K-response signatures were from refs. ^25,26^. Signature analysis was conducted as described^7^. Hierarchical clustering and heatmaps were generated using gplots package. Other plots were generated using ggplot2. log-rank tests of survival curve difference were calculated using survival package. A multivariate Cox proportional hazards model was built using the coxph function (Extended Data Fig. 6h). Significance of overlap was calculated using chisq.test or fisher.test function.

### Reporting summary

Further information on research design is available in the Nature Research Reporting Summary linked to this paper.

## Online content

Any methods, additional references, Nature Research reporting summaries, source data, extended data, supplementary information, acknowledgements, peer review information; details of author contributions and competing interests; and statements of data and code availability are available at 10.1038/s41586-020-2969-2.

## Supplementary information

Supplementary Information Supplementary Notes 1-4.

Life Sciences Reporting Summary

Supplementary Figure 1 Uncropped raw western blot images.

Supplementary Table 1 Basal-like breast cancer cell line and barcode information.

Supplementary Table 2 Metastatic potential of basal-like breast cancer.

Supplementary Table 3 MetMap cell line, pooling scheme, and updated annotation.

Supplementary Table 4 Metastatic potential in MetMap500.

Supplementary Table 5 Metastatic potential in MetMap125.

Supplementary Table 6 Signature gene list of Chr 8p genes, PI3K-response signatures, and brain stroma indicator.

Supplementary Table 7 Differential analysis of RNA-Seq of SREBF1-KO versus -WT cells.

Supplementary Table 8 Differential analysis of RNA-Seq of *in vivo* metastases versus *in-silico*-modeled *in vitro* profiles from breast cancer pools.

Supplementary Table 9 Tissue dissociation buffers and programs.

Supplementary Table 10 Guide sequences used for gene perturbation in CRISPR screen and validation; data count of mini-pool screen; CRISPR-seq primers.

## Data Availability

MetMap data and interactive visualization can be accessed at https://pubs.broadinstitute.org/metmap. RNA-seq data generated from this study have been deposited in the Gene Expression Omnibus (GEO) under accession numbers GSE148283 and GSE148372. Additional datasets used in this study include METABRIC, TCGA and MSK-targeted-sequencing breast cancer datasets from cBioPortal, the EMC-MSK dataset (GSE2035, GSE2603, GSE5327 and GSE12276), the 65-metastasis-sample dataset (GSE14020), paired primary tumour and brain metastasis RNA-seq from ref. ^36^, and GSE52604. Source data are provided with this paper.

## Source data

Source Data Fig. 1
Source Data Fig. 2
Source Data Fig. 3
Source Data Fig. 4
Source Data Fig. 5
Source Data Extended Data Fig. 1
Source Data Extended Data Fig. 2
Source Data Extended Data Fig. 3
Source Data Extended Data Fig. 4
Source Data Extended Data Fig. 5
Source Data Extended Data Fig. 6
Source Data Extended Data Fig. 7
Source Data Extended Data Fig. 8
Source Data Extended Data Fig. 9
Source Data Extended Data Fig. 10

## Code availability

Custom codes used for this study are accessible at the MetMap portal (https://pubs.broadinstitute.org/metmap).
