# Publisher Correction: A metastasis map of human cancer cell lines

**DOI:** 10.1038/s41586-021-04149-z

**Published:** 2021-11-03

**Authors:** Xin Jin, Zelalem Demere, Karthik Nair, Ahmed Ali, Gino B. Ferraro, Ted Natoli, Amy Deik, Lia Petronio, Andrew A. Tang, Cong Zhu, Li Wang, Danny Rosenberg, Vamsi Mangena, Jennifer Roth, Kwanghun Chung, Rakesh K. Jain, Clary B. Clish, Matthew G. Vander Heiden, Todd R. Golub

**Affiliations:** 1 Broad Institute of MIT and Harvard, Cambridge, MA USA; 2 Koch Institute for Integrative Cancer Research, Department of Biology, Massachusetts Institute of Technology, Cambridge, MA USA; 3 Edwin L. Steele Laboratories, Department of Radiation Oncology, Massachusetts General Hospital, Boston, MA USA; 4 Institute for Medical Engineering and Science, Picower Institute for Learning and Memory, Department of Chemical Engineering, Massachusetts Institute of Technology, Cambridge, MA USA; 5 Harvard Medical School, Boston, MA USA; 6 Dana-Farber Cancer Institute, Boston, MA USA

**Keywords:** Breast cancer, Cancer genomics, Cancer models, Metastasis

Correction to: *Nature* 10.1038/s41586-020-2969-2 Published online 9 December 2020

This paper was originally published under standard Springer Nature license (© The Author(s), under exclusive licence to Springer Nature Limited). It is now available as an open-access paper under a Creative Commons Attribution 4.0 International license, © The Author(s). The error has been corrected in the online version of the article.

