# Supplementary material for: A metastasis map of human cancer cell lines
Source: Nature. 2020 Dec 9;588(7837):331–6. doi: 10.1038/s41586-020-2969-2 (PMC8439149; doi:10.1038/s41586-020-2969-2)
Supplement: Supplementary file 1 — Supplementary Notes 1-4. [file 41586_2020_2969_MOESM1_ESM.pdf]

nature portfolio

https://doi.org/10.1038/s41586-020-2969-2

Supplementary information

A metastasis map of human cancer cell lines

In the format provided by the authors and unedited

Supplementary Notes for

MetMap: a map of metastatic potential of human cancer cell lines

Xin Jin [1,*], Zelalem Demere [1], Karthik Nair [1], Ahmed Ali [1,2], Gino B. Ferraro [3], Ted Natoli [1], Amy Deik [1], Lia Petronio [1], Andrew A. Tang [1], Cong Zhu [1], Li Wang [1], Danny Rosenberg [1], Vamsi Mangena [4], Jennifer Roth [1], Kwanghun Chung [1,4], Rakesh K. Jain [3,5], Clary B. Clish [1], Matthew G. Vander Heiden [1,2,6], Todd R. Golub [1,5,6,*]

1. Broad Institute of MIT and Harvard, Cambridge, MA, USA
2. Koch Institute for Integrative Cancer Research, Department of Biology, Massachusetts Institute of Technology, Cambridge, MA, USA
3. Edwin L. Steele Laboratories, Department of Radiation Oncology, Massachusetts General Hospital, Boston, MA, USA
4. Institute for Medical Engineering and Science, Picower Institute for Learning and Memory, Department of Chemical Engineering, Massachusetts Institute of Technology, Cambridge, MA, USA
5. Harvard Medical School, Boston, MA, USA
6. Dana-Farber Cancer Institute, Boston, MA, USA

* Co-corresponding author

Supplementary Note 1. Scalable metastatic potential profiling with barcoded cell line pools.

To enable profiling of *in vivo* metastatic potential in a scalable manner (Fig. 1a, Extended Data Fig. 1), we designed a barcoding vector that contained (1) a fluorescence protein (GFP or mCherry) for cell sorting, (2) a luciferase for real-time *in vivo* imaging, and (3) a barcode for cell line identity tracing. The three elements constituted a single transcription cassette; thus their expression levels were correlated. This ensured that the labeled cell lines harbor close expression levels (and thus similar copy numbers) of barcodes through gating the fluorescence expression by FACS (Extended Data Fig. 1e). The designed barcodes could be readout at either DNA or RNA level, by TaqMan assay or by next-generation sequencing, suitable for both low-throughput and high-throughput applications.

The transcribing barcode design allows for co-capturing of cancer barcodes and cancer transcriptomes of metastases from bulk RNA-Seq, so we developed a workflow and analysis method that readout both (Fig. 1a). The resultant transcriptomic profiles represent an ensemble from multiple constituent cell lines, and yield consensus gene programs and generalizable molecular insights about organ-specific metastases. An example of the barcode mapping result from the pilot experiment is presented in Extended Data Fig. 1f. The barcodes are expressed at high levels, among the top 10% highly expressed genes, allowing robust quantification (Extended Data Fig. 1g,h).

To validate RNA-Seq-quantitated barcode results from the pilot study, we performed RT-qPCR using Taqman assays against the barcodes. An examination of individual barcoded lines showed that the Taqman probes were highly specific to the engineered barcodes and there was no cross detection (Extended Data Fig. 1i). Consistent with RNA-Seq (Extended Data Fig. 1d), RT-qPCR showed even distribution of 4 cell lines in the pre-injected pool, but selective enrichment of specific cell lines in different organs (Extended Data Fig. 1j). To further validate at single cell resolution, we performed single cell RNA-Seq on the isolated cancer cells from different organs, one organ per 96-well plate (Extended Data Fig. 1k). Principal component analysis (PCA) stratified cells into 2 clusters. One cluster was characterized by high expression of genes on the HER2 amplicon (ERBB2, ORMDL3, GRB7, PGAP3), consistent with the HCC1954 (HER2+) identity (Extended Data Fig. 1k,l). The other cluster was characterized by high expression of VIM (vimentin) and low expression of CDKN2A (P16), consistent with MDAMB231 harboring P16-loss and being vimentin-high (Extended Data Fig. 1k,l). Mapping cell line identities to their organ origins indicated that HCC1954 was abundant in the brain, whereas MDAMB231 dominated lung, liver and bone

(Extended Data Fig. 1m). In both approaches, BT549 and CAL851 were not detected. Collectively, these results validated the pilot study with independent methods.

Having validated the feasibility of *in vivo* barcoding approach, we set out to map the metastatic behaviors of basal-like breast cancers from Cancer Cell Line Encyclopedia (CCLE), which display substantial heterogeneity in metastatic patterns from patient to patient. Principal component analysis (PCA) of expression profiles stratified breast cancer cell lines into 3 categories: (1) one group all initiated with HS and displaying fibroblast characteristics, (2) one enriched in luminal subtype, and (3) one enriched in basal subtype (Extended Data Fig. 1a). Since 8 barcoded lines could be pooled without obvious bottleneck from the pilot study, we surmised a pool size of 10 would be suitable, and split the additional 17 lines into 2 pools (group1 and group2, Extended Data Fig. 1a). The two non-metastatic lines BT549 and CAL851 were included again in these two larger pools for re-assessment. Cell lines were individually barcoded (Supplementary Table 1), pooled at equal numbers, and injected into mice. Bioluminescence imaging indicated comparable tumor progression kinetics as the pilot experiment (Extended Data Fig. 1b), and thus all mice were sacrificed 5 weeks after injection, in a time-matched manner. The total cell numbers and barcode-quantitated cell line compositions from each organ sample are presented in Extended Data Fig. 1c,d.

To quantify the cell line metastatic potentials on an absolute scale, we inferred the cell number for each cell line based on the total cancer cell counts and their barcode-quantitated compositions from each organ. We used this metric to compare cell lines across the 3 pool studies (Supplementary Table 2). For data visualization, we developed a petal plot that encodes 3 metrics: (1) metastatic potential as quantified by inferred cell number, (2) its confidence interval that estimates animal variability, (3) and penetrance – percentage of animals in the cohort that the particular cell line is detected (Fig. 1d). This visualization method effectively displayed the diversity of metastatic patterns and differential aggressiveness of cell lines. Four cell lines including MDAMB231, HCC1187, JIMT1, HCC1806 were pan-metastatic. Other cell lines showed more selective patterns. Among the 21 cell lines, DU4475 and HCC1599 were suspension cells and both displayed selective colonization towards bone and lung. Whether the *in vivo* pattern is associated with cell culture status remains unclear.

Supplementary Note 2. Drafting MetMap with PRISM cell line pools.

We attempted to expand metastatic potential mapping beyond breast cancer and to draft a comprehensive MetMap for all solid tumor types. Focusing on one cancer type at a time would

result in custom pooling and different group sizing, which was neither scalable nor standardizable. For pan-cancer characterization, it also didn't make sense to perform bulk RNA-Seq on mixed cancer types, as lineage would be a strong confounder. In this case, readout at the DNA level would be sufficient. We thus resorted to PRISM, a barcoded cell line mixture approach developed for high-throughput *in vitro* drug screening, and asked whether the PRISM platform could be applied for the *in vivo* MetMap purpose.

As part of PRISM profiling, cell lines were pooled based on their *in vitro* doubling time across mixed lineages, with a size of 25 lines per pool (Supplementary Table 3). PRISM barcoded cells did not harbor GFP or luciferase, thus in the first study, we addressed whether it was critical to introduce the labeling markers for cancer cell purification. We chose one PRISM pool (of 25 cell lines) that contained JIMT1, labeled with GFP-luciferase vector, and then sorted for GFP+ cells (Extended Data Fig. 2a). Consistent with different susceptibilities of cell lines to virus infection, 6/25 cell lines showed strong dropout after GFP labeling, but all lines were still detectable (Extended Data Fig. 2b). In contrast, cell lines prior to labeling displayed a more even barcode distribution, close to equal ratio pooling. The GFP-labeled and unlabeled cell pools were then subjected to the same animal workflow, tissue dissociation, and mouse cell depletion. The GFP-labeled group was further sorted to purify cancer cells. The isolated cancer cells (GFP-labeled group) or the tissue lysates (unlabeled group) were then subjected to barcode amplification and sequencing (Extended Data Fig. 2a). A comparison of the two experiments showed highly concordant results. Although the initial barcode distribution of the pre-injected pools had altered, the enrichment (fold change) of barcode abundance showed strong positive correlation after normalizing to the respective pre-injected input (Extended Data Fig. 2c, one exception U2OS). The positive control JIMT1 was pan-metastatic as expected. Importantly, cell lines such as MELHO, MHHES1 and PC14 substantially dropped in their initial abundance after GFP labeling, yet they gained similar *in vivo* enrichment as in the non-labeled experiment. These results suggested that we could quantitatively detect barcodes from crude lysates without the need of pure cancer cell isolation from PRISM.

We thus employed the simplified workflow using PRISM pools for pan-cancer mapping, and profiled a total of 503 cancer cell lines across 21 cancer types (Fig. 2a). Profiling was carried out in two different pooling formats (MetMap500 and MetMap125), with 120 cell lines and 4 target organs shared in common that allowed reproducibility assessment (Fig. 2b). Prior to injection, cell lines displayed an even barcode distribution, consistent with equal ratio pooling (Extended Data Fig. 2d). In MetMap500, 10 cell lines had low initial abundance and could not be detected in any *in vivo* organ; they were thus excluded from analysis, leaving data for 488 cell lines (Supplementary Table 3). PRISM sequencing detected relative barcode abundance which was reflective of relative cell

abundance in organs. We thus defined metastatic potential as the enrichment of barcodes in the *in vivo* organs relative to the pre-injected input, and used this metric to compare between cell lines (Supplementary Table 4, 5). A comparison of normalized with non-normalized barcode counts showed strong linearity (Extended Data Fig. 2e), reflecting that subtle differences in the initial abundance had little impact on barcode quantification from *in vivo* samples. We employed a similar petal plot view to display metastatic patterns, including relative metastatic potential as readout by PRISM barcode, its confidence interval that depicts animal variability, and penetrance data that provides qualitative measures of cell line xenograftability (see MetMap portal at pubs.broadinstitute.org/metmap).

Supplementary Note 3. Analysis of *in vivo* metastasis transcriptomes with multiplexed cell line compositions.

As stated in the Main Text and Supplementary Note 1, RNA-Seq co-captured cancer cell composition and averaged *in vivo* transcriptomes of metastases from cell line pools in the breast cancer cohort study. To understand what metastasis transcriptomes encoded, we performed differential analysis on the *in vivo* transcriptomes versus cells *in vitro*. To properly account for the different cell line compositions in each metastasis, a composite *in vitro* transcriptome was modeled using the barcode composition and single cell line *in vitro* profiles, and then compared to the actual *in vivo* results (Extended Data Fig. 8a). In this way, the resultant differentially expressed genes were uniquely attributed to the *in vivo* context but not due to cell composition differences. These genes were (1) either commonly induced (or selected for) in multiple cell lines, or (2) were uniquely enriched in the dominant line. In either case, the revealed genes or pathways would be interesting for further study. As expected, the transcriptomes of the pre-injected population which was a direct mixture of *in vitro* cell lines showed a very tight correlation with the *in silico* profiles, and few genes were differentially expressed (Extended Data Fig. 8b). In contrast, the transcriptomes from *in vivo* samples showed genes with large fold changes and the correlation was weaker. These results justified the comparison method and showed that the *in vivo* environment was inducing substantial transcriptional changes. Supplementary Table 8 lists the detailed differential comparison analysis.

To assess whether such comparison identified genes relevant to metastasis, we inspected the top differentially expressed genes. Notably, MUCL1 (also termed small breast epithelial mucin, SBEM) and SCGB2A2 (also known as Mammaglobin, MGB1) were strongly induced in brain metastases as well as in other sites (Extended Data Fig. 8c). These genes are breast lineage markers, whose expression is known to be induced during breast tumorigenesis from clinical specimens. Their expression has been used as a marker of hematogenous spread, micrometastasis [1,2], and breast

cancer metastasis in the brain differentiating from primary brain tumors [3]. These results revealed that although relevant marker genes were lowly or non-expressed when cells were cultured in a dish, their expression could be induced in the *in vivo* metastasis context. These results highlighted the biological relevance of the *in vivo* transcriptomic results.

Since MDAMB231 is the most investigated cell line in breast cancer metastasis, we asked whether genes previously identified and validated as metastasis mediators were induced in the *in vivo* transcriptomic profiles. In the pilot group experiments, MDAMB231 dominated lung, liver, kidney and bone metastases in most samples (Extended Data Fig. 1d); thus the majority of the gene expression changes were attributed to MDAMB231. Twenty-seven out of 32 lung metastasis genes reported by Minn et al. [4] were upregulated in our lung metastasis profiles, showing a very strong agreement (p value = 3.9e-16, Extended Data Fig. 8d). These genes were also enriched in metastases at other sites but to a lesser extent. Indeed, although these genes were initially identified as lung metastasis mediators, many were shown to function in a pleiotropic fashion, mediating primary tumor or metastasis growth at other sites. For example, VCAM1 has been shown to mediate both lung and bone metastasis through juxtacrine interaction with myeloid lineage cells [5,6]. TNC, which is a secreted molecule that boosts breast cancer stemness, promotes lung and bone metastasis [7]. Collectively, these results suggested that the *in vivo* “induced” genes not only included metastasis associated markers but also functional mediators.

Having confirmed the validity of these profiles, we performed pathway enrichment analysis [8] to query consensus programs that the differential genes encode at the 5 organ sites. The results revealed a diverse *in vivo* response to external stimuli, suggestive of richer environmental factors in the animal (Extended Data Fig. 8f). In contrast, proliferation and cycling pathways are much attenuated *in vivo* compared to *in vitro* cells. Consistent with this result, *in vitro* culture media is optimized for maximal cell proliferation by supplementing excess nutrients and supportive elements [9]. Comparing between organs, we found that brain metastases shared less commonality and weaker correlation with metastases in other organs (Extended Data Fig. 8e), suggesting a more unique microenvironment in the brain. More specifically, inflammatory responses including TNF, interleukin and interferon signaling were more prominent in lung, liver, kidney, bone than in brain, consistent with less immune response in the brain compared to extracranial organs [10]. Similarly, we saw evidence of TGFβ activation and epithelial-mesenchymal transition (EMT) in extracranial metastatic lesions, but not in brain (Extended Data Fig. 8f). In contrast, brain is uniquely enriched in lipid metabolism related pathways (Extended Data Fig. 8f-i). Confirming these experimental observations, brain metastasis samples from patients showed less TGFβ, EMT responses and enriched expression of lipid metabolism genes, in comparison to extracranial metastases or

matched primary breast tumors (Extended Data Fig. 9). Together, these results revealed distinct cell transcriptional states between *in vitro* and *in vivo*, and between different metastasis sites.

Supplementary Note 4. Mini-pool *in vivo* CRISPR screen in brain metastasis.

To interrogate the importance of lipid metabolism in brain metastasis, we performed a CRISPR screen of 29 genes in brain metastasis using the JIMT1 cell model. We adapted the workflow of *in vivo* PRISM to readout relative fitness of different gene perturbations by enumerating CRISPR guide abundance from tissue (Fig. 5a). Of note, no control guides were included in the pool as the presence of wild-type cells would dominate and limit the resolution of distinguishing between very strong hits. To restrict the phenotype to post-seeding events and focus on cellular adaptation to the brain microenvironment, we introduced cells through intracranial injection (Fig. 5a). The results revealed 13 significant genes which included SREBF1, SCAP, and SCD (FDR < 0.05, Fig. 5b). In addition, 2 mevalonate/cholesterol pathway genes, PMVK and UBIAD1 showed deepest *in vivo* depletion (Fig. 5b).

We selected 6 genes for individual validation, and all 6 resulted in a strong brain metastasis defect (Fig. 5c). In contrast to the exponential growth of wild-type cells following injection, SREBF1-knock-out cells showed minimal increase in tumor burden (Fig. 5c). Perturbing genes upstream and downstream of SREBF1, SCAP and SCD respectively, phenocopied the SREBF1-knock-out effect and restricted cell proliferation in the brain. Mice displayed a minimal but detectable signal. Knocking out PMVK regressed the injected tumor cells and animals were signal-free, validating it as the strongest hit from the screen. Together, these results pinpointed the significance of lipid and cholesterol metabolism in mediating brain metastasis outgrowth.

In order to understand the generality of these findings, we further assessed these 6 genes in an independent cell model HCC1806 (Extended Data Fig. 10d). Brain metastatic growth was inhibited by all the 6 gene knock-outs, but the magnitude of effect was smaller for SREBF1, SCAP, and SCD. The results indicated that there was an SREBF-independent mechanism in place to support regrowth of HCC1806 in the brain (Extended Data Fig. 10e-i). PMVK-knock-out was the exception that resulted in complete tumor cell regression similarly as observed in JIMT1 (Extended Data Fig. 10d). These results highlighted both selectivity and generality of the lipid metabolism gene dependencies in brain metastasis.

References for Supplementary Notes

1. Cerveira, N. *et al.* Highly sensitive detection of the MGB1 transcript (mammaglobin) in the peripheral blood of breast cancer patients. *Int J Cancer* **108**, 592-595 (2004).
2. Valladares-Ayerbes, M. *et al.* Diagnostic accuracy of small breast epithelial mucin mRNA as a marker for bone marrow micrometastasis in breast cancer: a pilot study. *J Cancer Res Clin Oncol* **135**, 1185-1195 (2009).
3. Cimino, P. J., Jr. & Perrin, R. J. Mammaglobin-A immunohistochemistry in primary central nervous system neoplasms and intracranial metastatic breast carcinoma. *Appl Immunohistochem Mol Morphol* **22**, 442-448 (2014).
4. Minn, A. J. *et al.* Genes that mediate breast cancer metastasis to lung. *Nature* **436**, 518-524 (2005).
5. Chen, Q., Zhang, X. H. & Massague, J. Macrophage binding to receptor VCAM-1 transmits survival signals in breast cancer cells that invade the lungs. *Cancer Cell* **20**, 538-549 (2011).
6. Lu, X. *et al.* VCAM-1 promotes osteolytic expansion of indolent bone micrometastasis of breast cancer by engaging alpha4beta1-positive osteoclast progenitors. *Cancer Cell* **20**, 701-714 (2011).
7. Oskarsson, T. *et al.* Breast cancer cells produce tenascin C as a metastatic niche component to colonize the lungs. *Nat Med* **17**, 867-874 (2011).
8. Liberzon, A. *et al.* The Molecular Signatures Database (MSigDB) hallmark gene set collection. *Cell Syst* **1**, 417-425 (2015).
9. Yao, T. & Asayama, Y. Animal-cell culture media: History, characteristics, and current issues. *Reprod Med Biol* **16**, 99-117 (2017).
10. Louveau, A., Harris, T. H. & Kipnis, J. Revisiting the Mechanisms of CNS Immune Privilege. *Trends Immunol* **36**, 569-577 (2015).
