# Supplementary material for: A metastasis map of human cancer cell lines
Source: Nature. 2020 Dec 9;588(7837):331–6. doi: 10.1038/s41586-020-2969-2 (PMC8439149; doi:10.1038/s41586-020-2969-2)
Supplement: Supplementary file 2 — Life Sciences Reporting Summary [file 41586_2020_2969_MOESM2_ESM.pdf]

nature research

Corresponding author(s): Todd R Golub; Xin Jin

Last updated by author(s): Jul 21, 2020

Reporting Summary

Nature Research wishes to improve the reproducibility of the work that we publish. This form provides structure for consistency and transparency in reporting. For further information on Nature Research policies, see Authors & Referees and the Editorial Policy Checklist.

Statistics

For all statistical analyses, confirm that the following items are present in the figure legend, table legend, main text, or Methods section.

n/a	Confirmed	
☐	☒	The exact sample size (*n*) for each experimental group/condition, given as a discrete number and unit of measurement
☐	☒	A statement on whether measurements were taken from distinct samples or whether the same sample was measured repeatedly
☐	☒	The statistical test(s) used AND whether they are one- or two-sided<br>*Only common tests should be described solely by name; describe more complex techniques in the Methods section.*
☐	☒	A description of all covariates tested
☐	☒	A description of any assumptions or corrections, such as tests of normality and adjustment for multiple comparisons
☐	☒	A full description of the statistical parameters including central tendency (e.g. means) or other basic estimates (e.g. regression coefficient) AND variation (e.g. standard deviation) or associated estimates of uncertainty (e.g. confidence intervals)
☐	☒	For null hypothesis testing, the test statistic (e.g. *F*, *t*, *r*) with confidence intervals, effect sizes, degrees of freedom and *P* value noted<br>*Give P values as exact values whenever suitable.*
☐	☒	For Bayesian analysis, information on the choice of priors and Markov chain Monte Carlo settings
☐	☒	For hierarchical and complex designs, identification of the appropriate level for tests and full reporting of outcomes
☐	☒	Estimates of effect sizes (e.g. Cohen's *d*, Pearson's *r*), indicating how they were calculated

Our web collection on statistics for biologists contains articles on many of the points above.

Software and code

Policy information about availability of computer code

Data collection	Bioluminescence imaging data was acquired with Living Image software (v4.5, PerkinElmer). Lipidomics mass spectrometry data was acquired using a LC-MS system composed of a Shimadzu Nexera X2 U-HPLC (Shimadzu Corp) coupled to a Exactive Plus orbitrap mass spectrometer (ThermoFisher Scientific).
Data analysis	Following softwares were used for data analysis: Living Image software (v4.5), Bowtie 2 (v2.2.8), samtools (v 1.3.1), BBSplit (https://sourceforge.net/projects/bbmap/), RSEM (v1.3.1), R statistical software (v3.6.2), ggplot2 (3.3.0), limma (3.42.2), edgeR (3.28.0), gsva (1.34.0), gplots (3.0.1.2), survival (3.1-8), fgsea (1.12.0), GSEA (v3.0), GenePattern (v2.0).

For manuscripts utilizing custom algorithms or software that are central to the research but not yet described in published literature, software must be made available to editors/reviewers. We strongly encourage code deposition in a community repository (e.g. GitHub). See the Nature Research guidelines for submitting code & software for further information.

Data

Policy information about availability of data

All manuscripts must include a data availability statement. This statement should provide the following information, where applicable:

- Accession codes, unique identifiers, or web links for publicly available datasets
- A list of figures that have associated raw data
- A description of any restrictions on data availability

MetMap data and interactive visualization can be accessed at pubs.broadinstitute.org/metmap. RNA-seq data generated from this study have been deposited to Gene Expression Omnibus (GEO), at accession numbers GSE148283 and GSE148372. Additional datasets used in this study include METABRIC, TCGA, and MSK-targeted-sequencing breast cancer datasets downloadable from cBioPortal, EMC-MSK dataset (GSE2035, GSE2603, GSE5327, GSE12276), 65 metastasis sample dataset (GSE14020), paired primary tumor and brain metastasis RNA-Seq from Vareslija et al, and GSE52604.

Field-specific reporting

Please select the one below that is the best fit for your research. If you are not sure, read the appropriate sections before making your selection.

☒ Life sciences ☐ Behavioural & social sciences ☐ Ecological, evolutionary & environmental sciences

For a reference copy of the document with all sections, see nature.com/documents/nr-reporting-summary-flat.pdf

Life sciences study design

All studies must disclose on these points even when the disclosure is negative.

Sample size	Sample sizes were determined to be adequate for minimal n required for statistical tests, or consistency of measurable differences between groups following guidance and experience from Ref. 5, 7, 18.
Data exclusions	Failed RNA-Seq samples were excluded from analysis presented in the manuscript. In MetMap500 experiment (Fig. 2), one animal died early and organs could not be collected in time, and is excluded from analysis.
Replication	Cell culture based experiments (including growth assay, RT-qPCR, western blot) were performed twice independently. Animal experiments were validated using completely independent methods instead of direct repeat (Pooled experiment vs individual injection in Fig. 1a, Extended Data Fig. 2g; MetMap500 vs MetMap125 in Fig. 2c; mini-pool CRISPR screen vs one-by-one testing in Fig. 5a-c).
Randomization	Randomization was not applicable to experiments in this study. In MetMap profiling, we varied pooling format, cell density, cohort size, animal age to account for these potential covariates.
Blinding	Blinding to group allocations was not applicable to experiments in this study.

Reporting for specific materials, systems and methods

We require information from authors about some types of materials, experimental systems and methods used in many studies. Here, indicate whether each material, system or method listed is relevant to your study. If you are not sure if a list item applies to your research, read the appropriate section before selecting a response.

Materials & experimental systems

n/a	Involved in the study	
☐	☒	Antibodies
☐	☒	Eukaryotic cell lines
☒	☐	Palaeontology
☐	☒	Animals and other organisms
☒	☐	Human research participants
☒	☐	Clinical data

Methods

n/a	Involved in the study	
☒	☐	ChIP-seq
☐	☒	Flow cytometry
☒	☐	MRI-based neuroimaging

Antibodies

Antibodies used	SREBF1 primary antibody (14088-1-AP, Proteintech)<br>SCD (CD.E10) antibody (ab19862, Abcam)<br>GAPDH (D16H11) XP® Rabbit mAb (5174S, Cell Signaling)<br>β-Actin (8H10D10) Mouse mAb (3700S, Cell Signaling)<br>IRDye® 800CW Goat anti-Mouse IgG (926-32210, LI-COR)<br>IRDye® 680RD Goat anti-Rabbit IgG secondary antibodies (926-68071, LI-COR).
Validation	SREBF1 primary antibody (14088-1-AP, Proteintech): validated by manufacturer, and by this study (Extended Data Fig. 11f,h), and cited in publications, suitable for western blot<br>SCD (CD.E10) antibody (ab19862, Abcam): validated by manufacturer, and by this study (Extended Data Fig. 11f,h), suitable for western blot<br>GAPDH (D16H11) XP® Rabbit mAb (5174S, Cell Signaling): validated by manufacturer and cited in publications, suitable for western blot<br>β-Actin (8H10D10) Mouse mAb (3700S, Cell Signaling): validated by manufacturer and cited in publications, suitable for western blot

Eukaryotic cell lines

Policy information about cell lines

Cell line source(s)	All cell lines listed in Supplementary Table 1 and 3 were obtained from CCLE.
Authentication	Cell lines were authenticated by DNA fingerprinting analysis. The breast cell line identities were also confirmed by RNA-Seq and compared to CCLE RNA-Seq profiles.
Mycoplasma contamination	All cell lines were confirmed to be mycoplasma free using the MycoAlertTM Mycoplasma Detection Kit (Lonza).
Commonly misidentified lines (See ICLAC register)	PC-14 was identical to PC-9 as reported before (https://web.expasy.org/cellosaurus/CVCL_1640; https://www.sigmaaldrich.com/catalog/product/sigma/cb_90071810?lang=en&region=US). To keep consistent with CCLE nomenclature, PC14_LUNG was used. KPL-1 was found to be a MCF-7 derivative (https://web.expasy.org/cellosaurus/CVCL_2094). To keep separate from MCF-7 and consistent with CCLE nomenclature, KPL1_BREAST was used.

Animals and other organisms

Policy information about studies involving animals; ARRIVE guidelines recommended for reporting animal research

Laboratory animals	NOD scid gamma (NSG) female mice (The Jackson Laboratory) of 5~6 or 8~10 weeks were used for metastasis xenograft studies. Broad Vivarium's housing conditions for NSG mice include sterilized, individually ventilated cages with cellulose bedding. Water bottles are supplied with acidified, reverse osmosis water. The holding room is maintained under positive pressure, temperature 70°F (+/-2°F), humidity 40% (+/- 10%), lighting 12 on/12 off light cycle.
Wild animals	No wild animals were used in the study.
Field-collected samples	No field collected samples were used in the study.
Ethics oversight	Animal work was performed in accordance with a protocol approved by the Broad Institute Institutional Animal Care and Use Committee (IACUC).

Note that full information on the approval of the study protocol must also be provided in the manuscript.

Flow Cytometry

Plots

Confirm that:

☒ The axis labels state the marker and fluorochrome used (e.g. CD4-FITC).

☒ The axis scales are clearly visible. Include numbers along axes only for bottom left plot of group (a 'group' is an analysis of identical markers).

☒ All plots are contour plots with outliers or pseudocolor plots.

☒ A numerical value for number of cells or percentage (with statistics) is provided.

Methodology

Sample preparation	Organs were dissociated using dissociation protocols listed in Supplementary Table 9 with gentleMACS Octo Dissociator (Miltenyi Biotec). Dissociated cell suspensions were filtered using 100 µm filters, and washed with DMEM/F12 twice. Cell suspensions were then washed with staining buffer (PBS + 2mM EDTA + 0.5% BSA), and incubated with mouse cell depletion beads according to the instructions (Miltenyi Biotec). Cell suspensions were subjected to negative selection using autoMACS Pro Separator (Miltenyi Biotec) to deplete mouse stroma. Brains were subjected to an additional myelin debri depletion step using myelin removal beads II (Miltenyi Biotec). In vitro cultured cells were trypsinized and resuspended as single cell suspensions. DAPI staining was used to exclude dead cells.
Instrument	SONY SH4800
Software	SH4800S and FlowJo (v10.2)
Cell population abundance	Data is presented in Extended Data Fig 1c and Source Data.
Gating strategy	Gating strategy is illustrated in Extended Data Fig. 1e to select for single cells with the fixed gate for GFP or mCherry.

☒ Tick this box to confirm that a figure exemplifying the gating strategy is provided in the Supplementary Information.
