# Supplementary material for: A metastasis map of human cancer cell lines
Source: Nature. 2020 Dec 9;588(7837):331–6. doi: 10.1038/s41586-020-2969-2 (PMC8439149; doi:10.1038/s41586-020-2969-2)
Supplement: Supplementary file 3 — Uncropped raw western blot images. [file 41586_2020_2969_MOESM3_ESM.pdf]

ED Fig. 11f

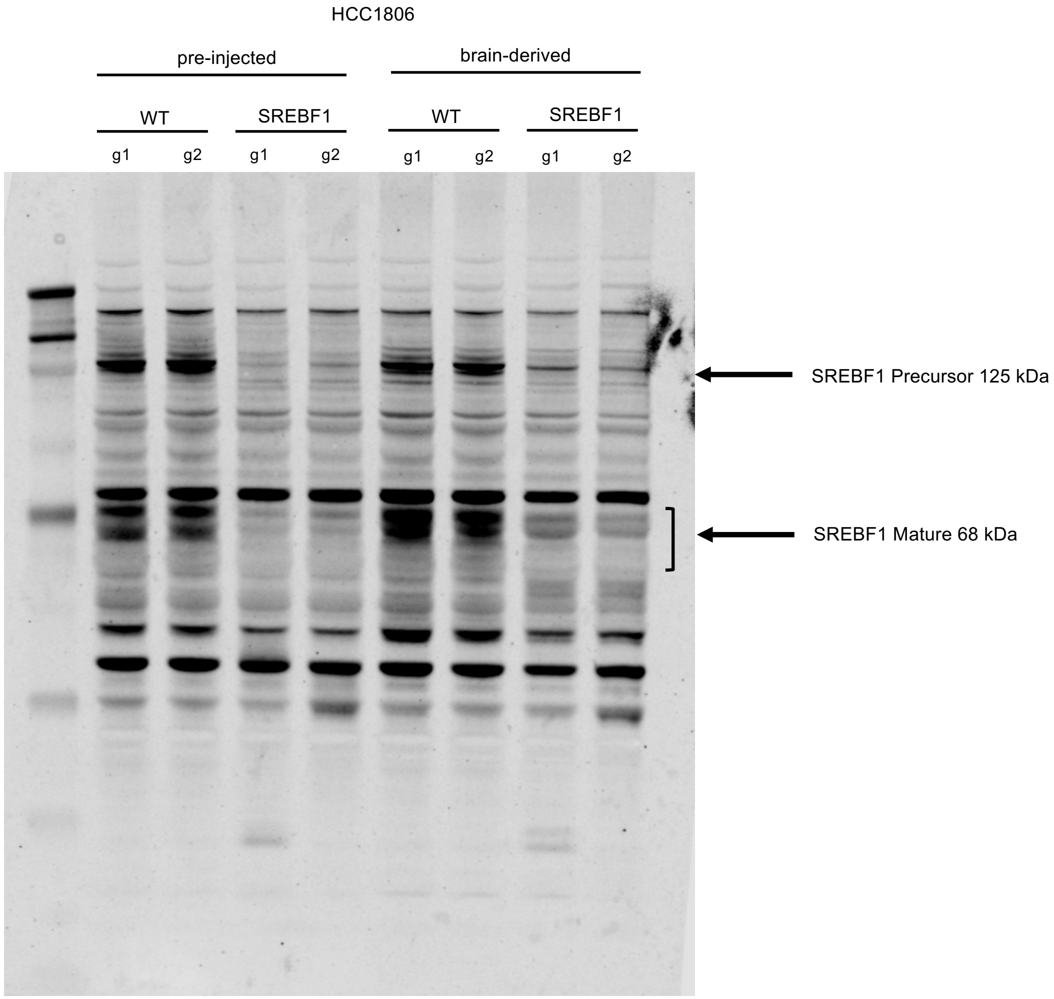
HCC1806
pre-injected
brain-derived
WT
SREBF1
WT
SREBF1
g1
g2
g1
g2
g1
g2
g1
g2
SREBF1 Precursor 125 kDa
SREBF1 Mature 68 kDa

ED Fig. 11f

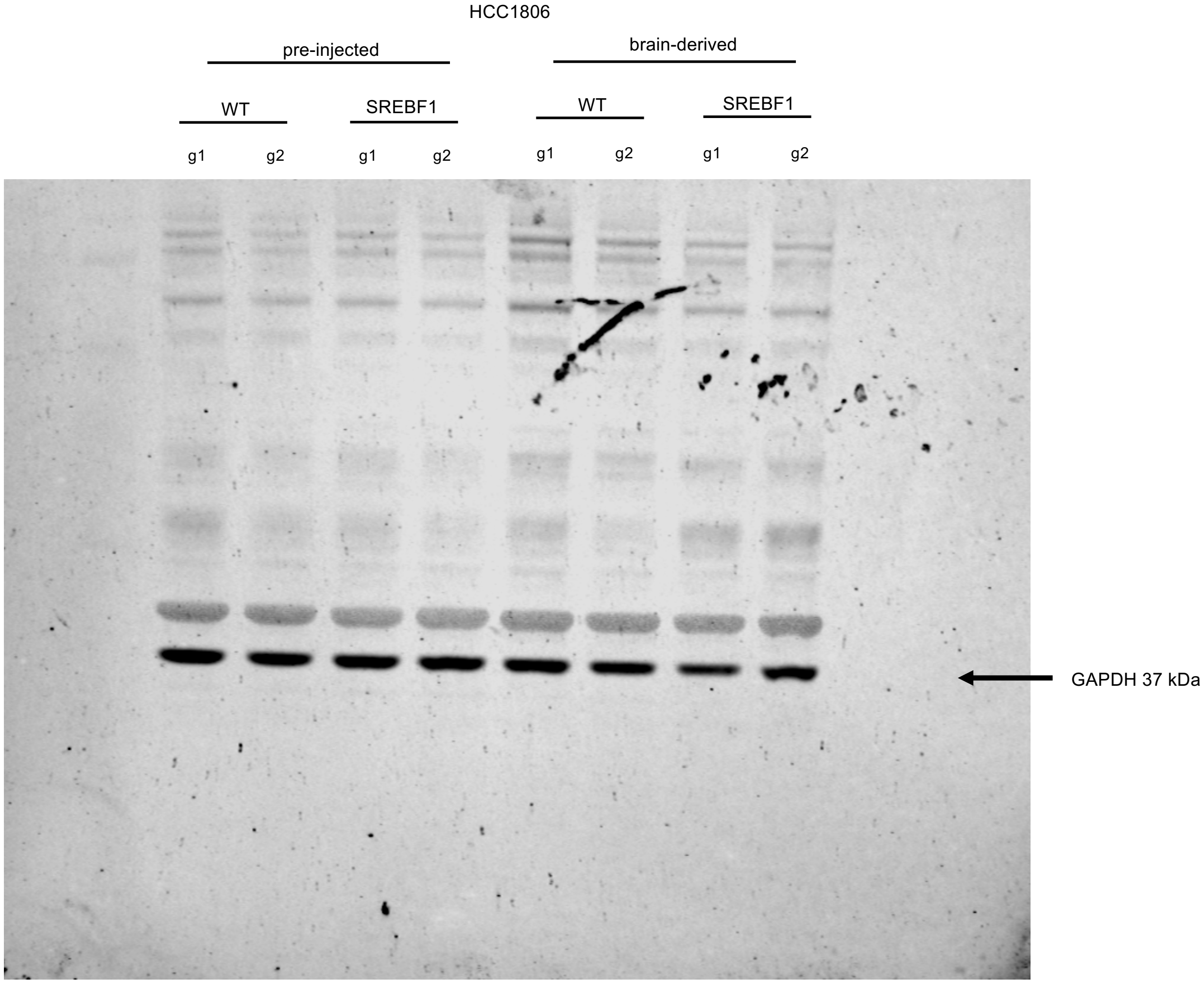

HCC1806
pre-injected
brain-derived
WT
SREBF1
WT
SREBF1
g1
g2
g1
g2
g1
g2
g1
g2
GAPDH 37 kDa

ED Fig. 11f

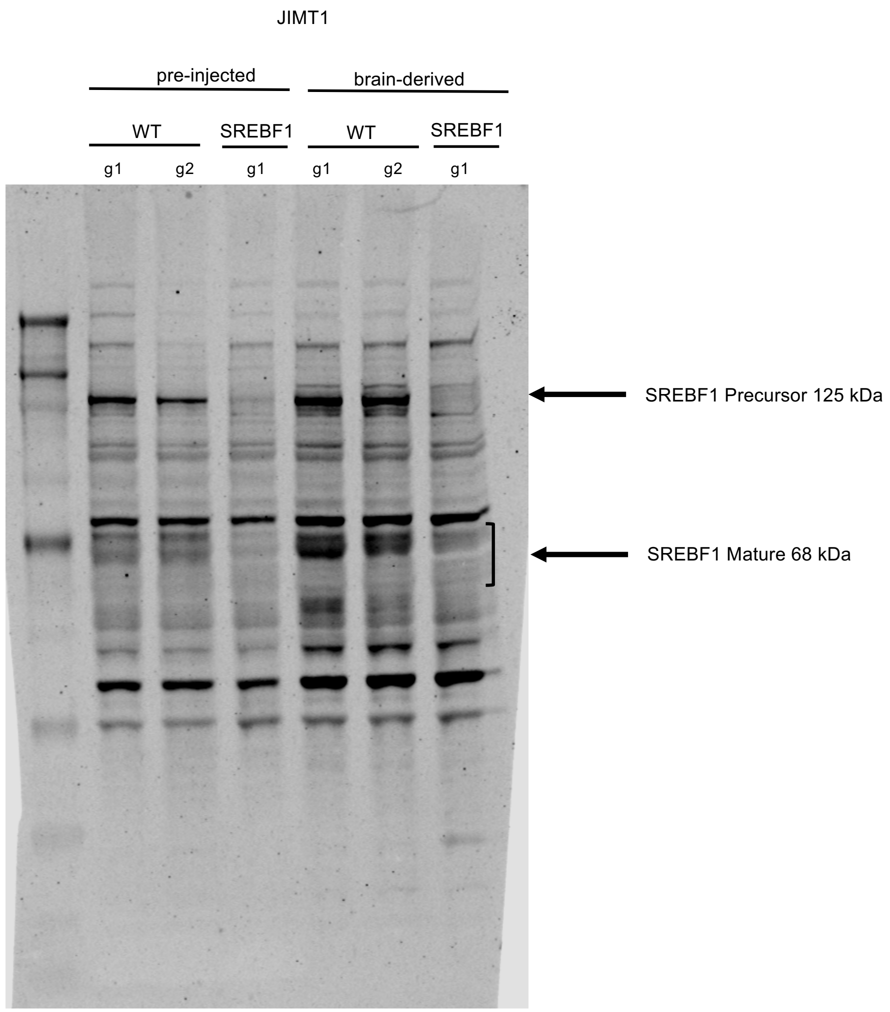

JIMT1
pre-injected
brain-derived
WT
SREBF1
WT
SREBF1
g1
g2
g1
g1
g2
g1
SREBF1 Precursor 125 kDa
SREBF1 Mature 68 kDa

ED Fig. 11f

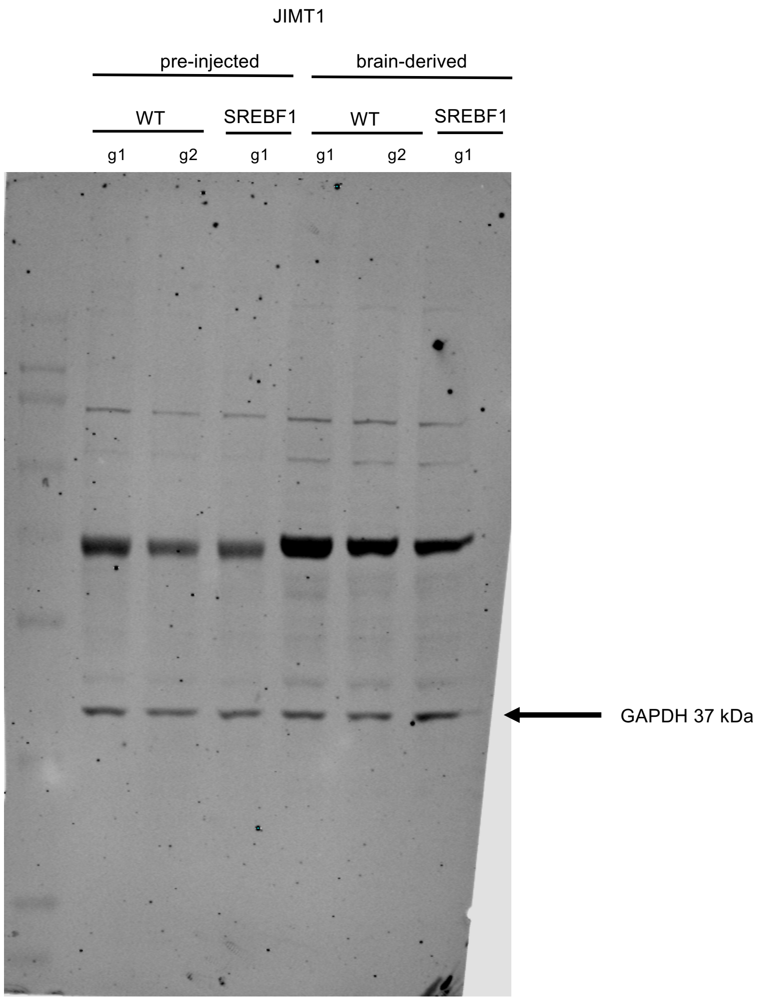
JIMT1
pre-injected
brain-derived
WT
SREBF1
WT
SREBF1
g1
g2
g1
g1
g2
g1
GAPDH 37 kDa

ED Fig. 11h

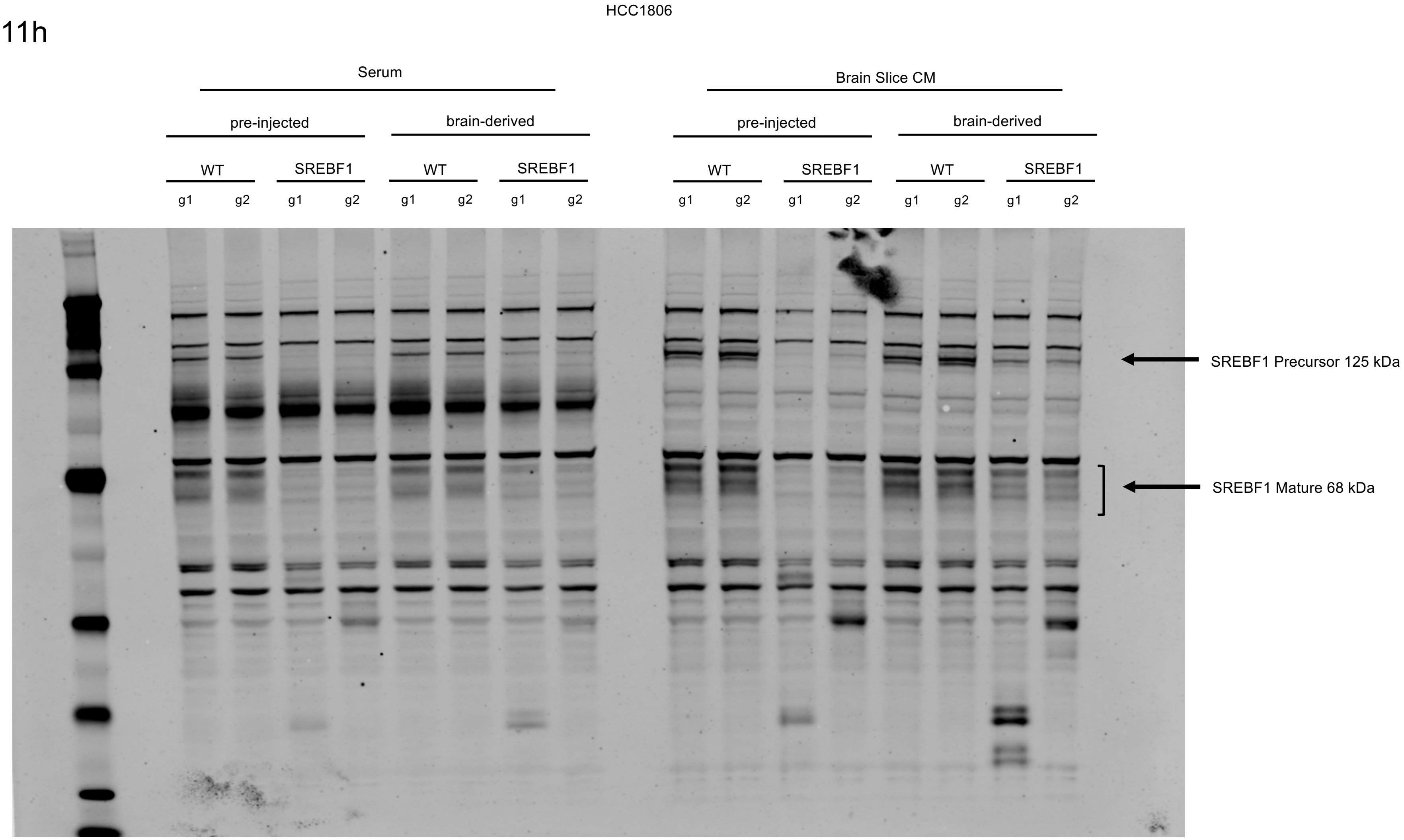
HCC1806
Serum
Brain Slice CM
pre-injected
brain-derived
pre-injected
brain-derived
WT
SREBF1
WT
SREBF1
WT
SREBF1
WT
SREBF1
g1 g2 g1 g2 g1 g2 g1 g2 g1 g2 g1 g2 g1 g2 g1 g2
SREBF1 Precursor 125 kDa
SREBF1 Mature 68 kDa

ED Fig. 11h

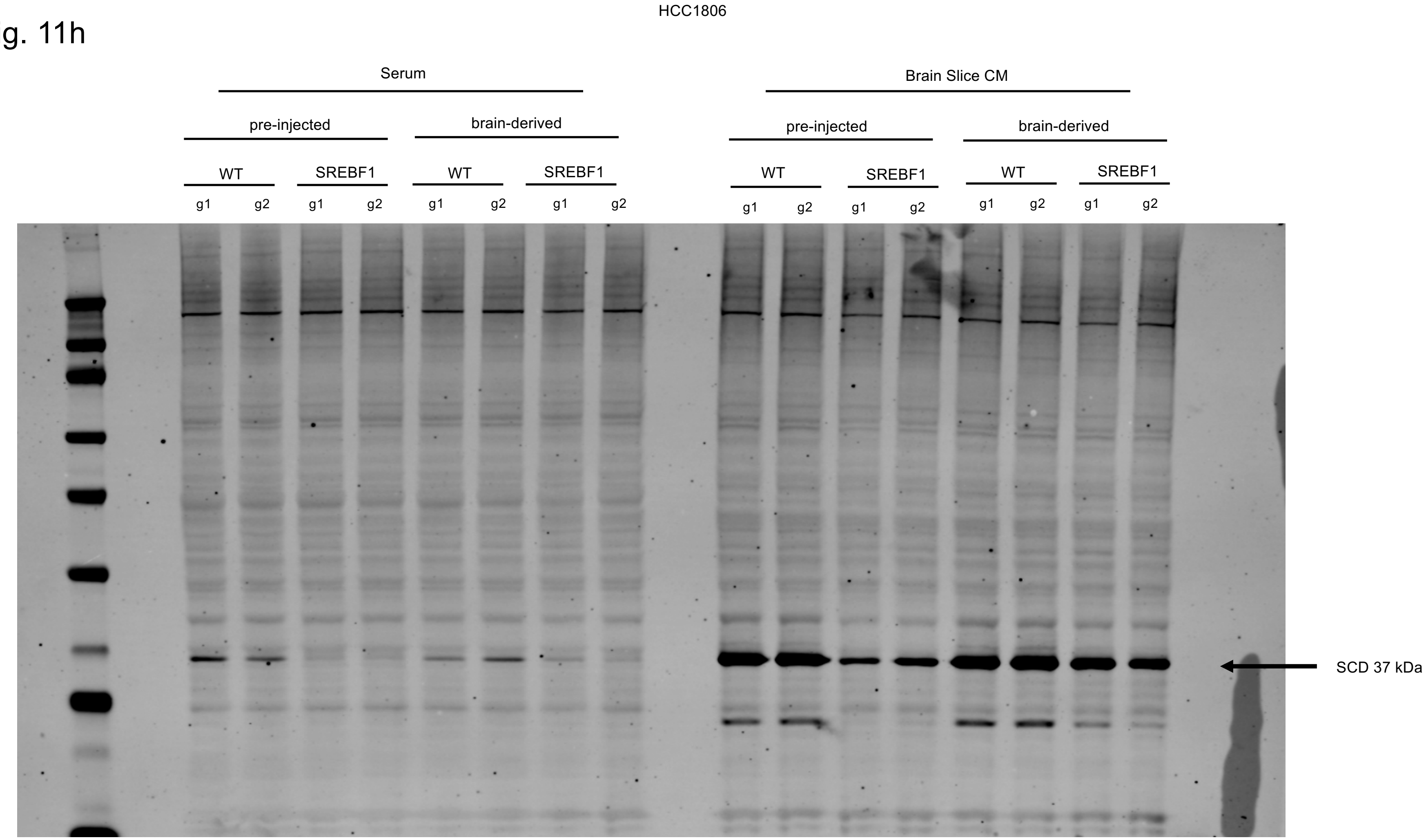
HCC1806
Serum
Brain Slice CM
pre-injected
brain-derived
pre-injected
brain-derived
WT
SREBF1
WT
SREBF1
WT
SREBF1
WT
SREBF1
g1 g2 g1 g2 g1 g2 g1 g2 g1 g2 g1 g2 g1 g2 g1 g2
SCD 37 kDa

ED Fig. 11h

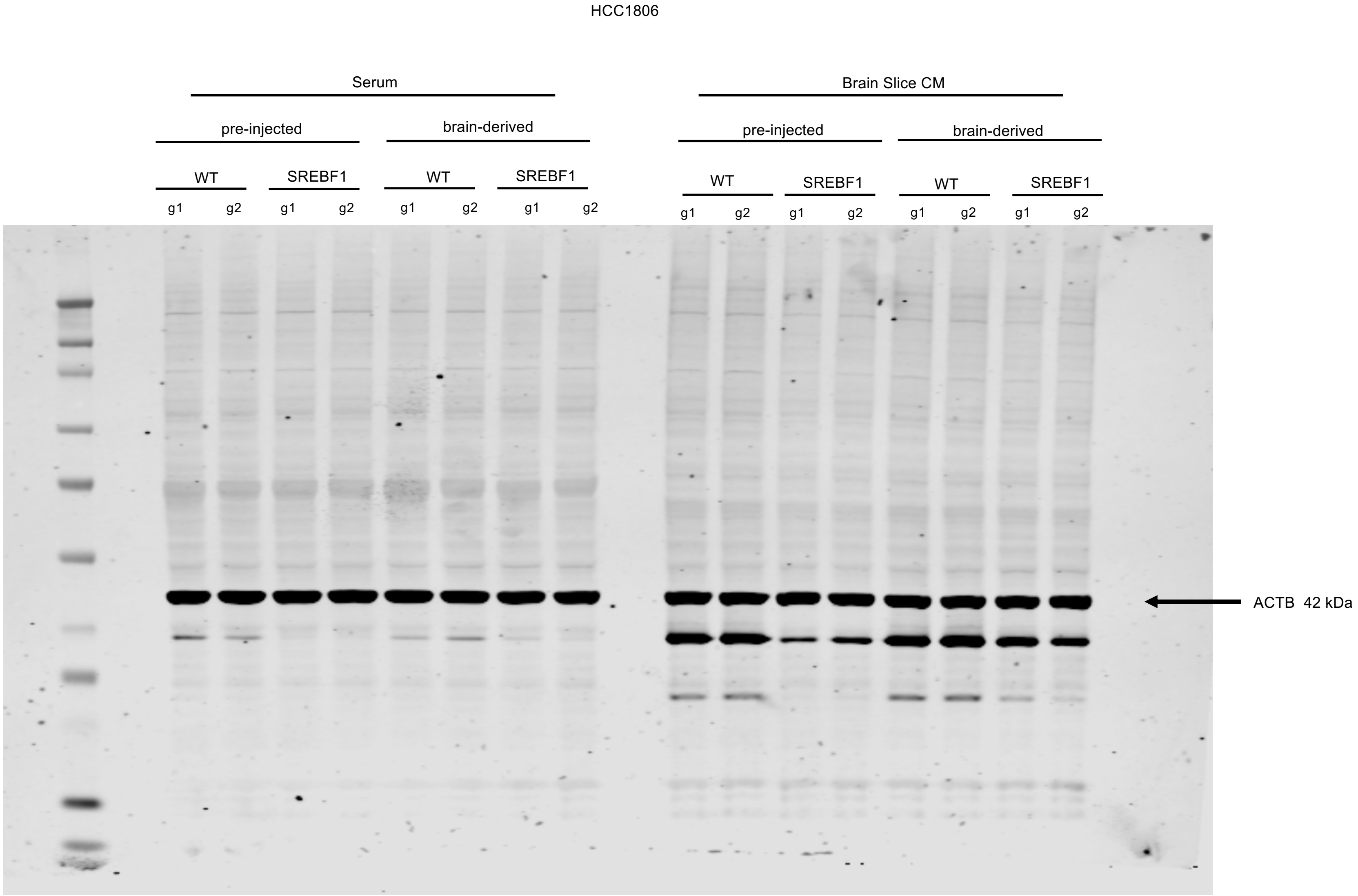
HCC1806
Serum
Brain Slice CM
pre-injected
brain-derived
pre-injected
brain-derived
WT
SREBF1
WT
SREBF1
WT
SREBF1
WT
SREBF1
g1 g2 g1 g2 g1 g2 g1 g2 g1 g2 g1 g2 g1 g2 g1 g2
ACTB 42 kDa

ED Fig. 11h

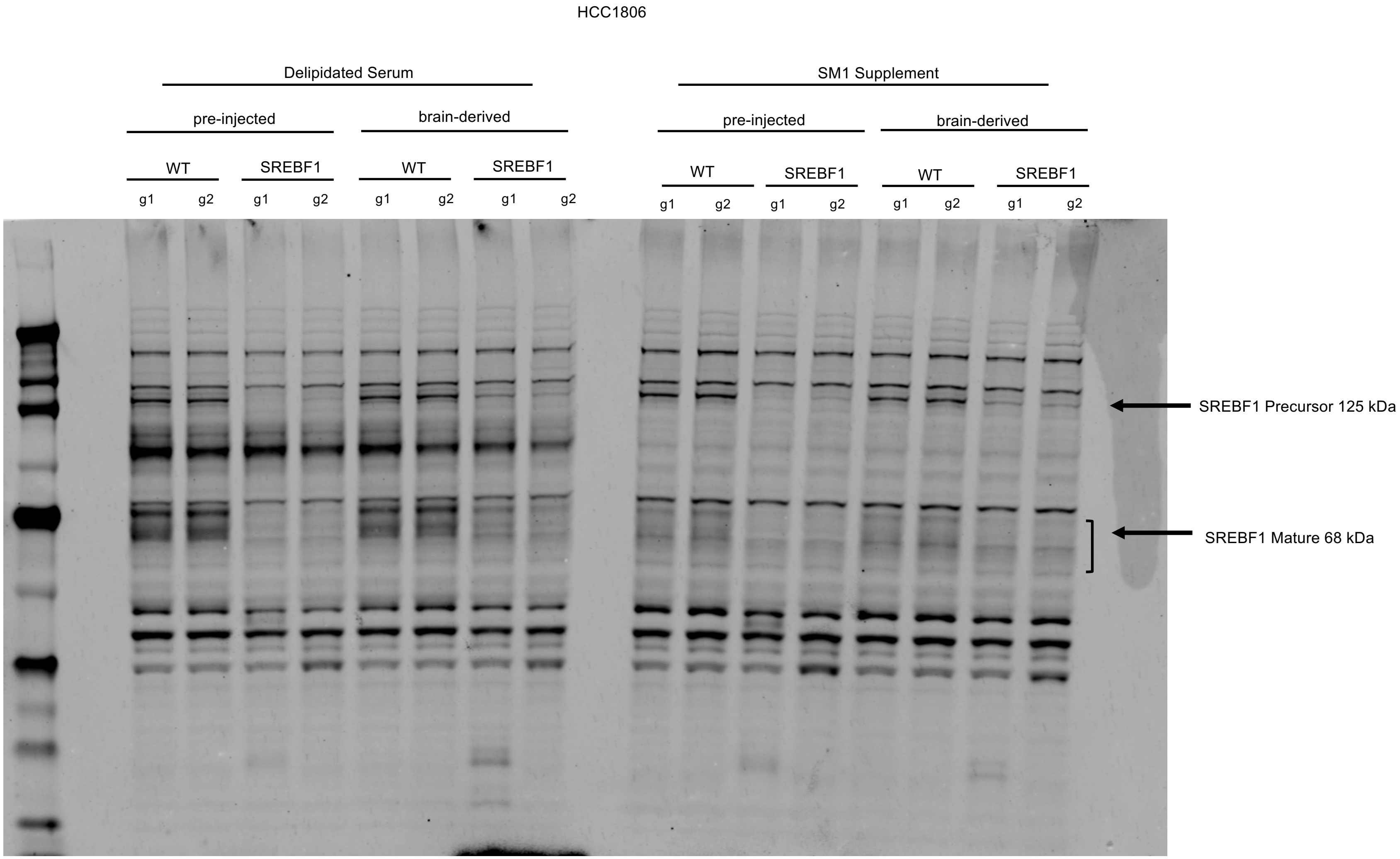
HCC1806
Delipidated Serum
SM1 Supplement
pre-injected
brain-derived
pre-injected
brain-derived
WT
SREBF1
WT
SREBF1
WT
SREBF1
WT
SREBF1
g1 g2 g1 g2 g1 g2 g1 g2 g1 g2 g1 g2 g1 g2 g1 g2
SREBF1 Precursor 125 kDa
SREBF1 Mature 68 kDa

ED Fig. 11h

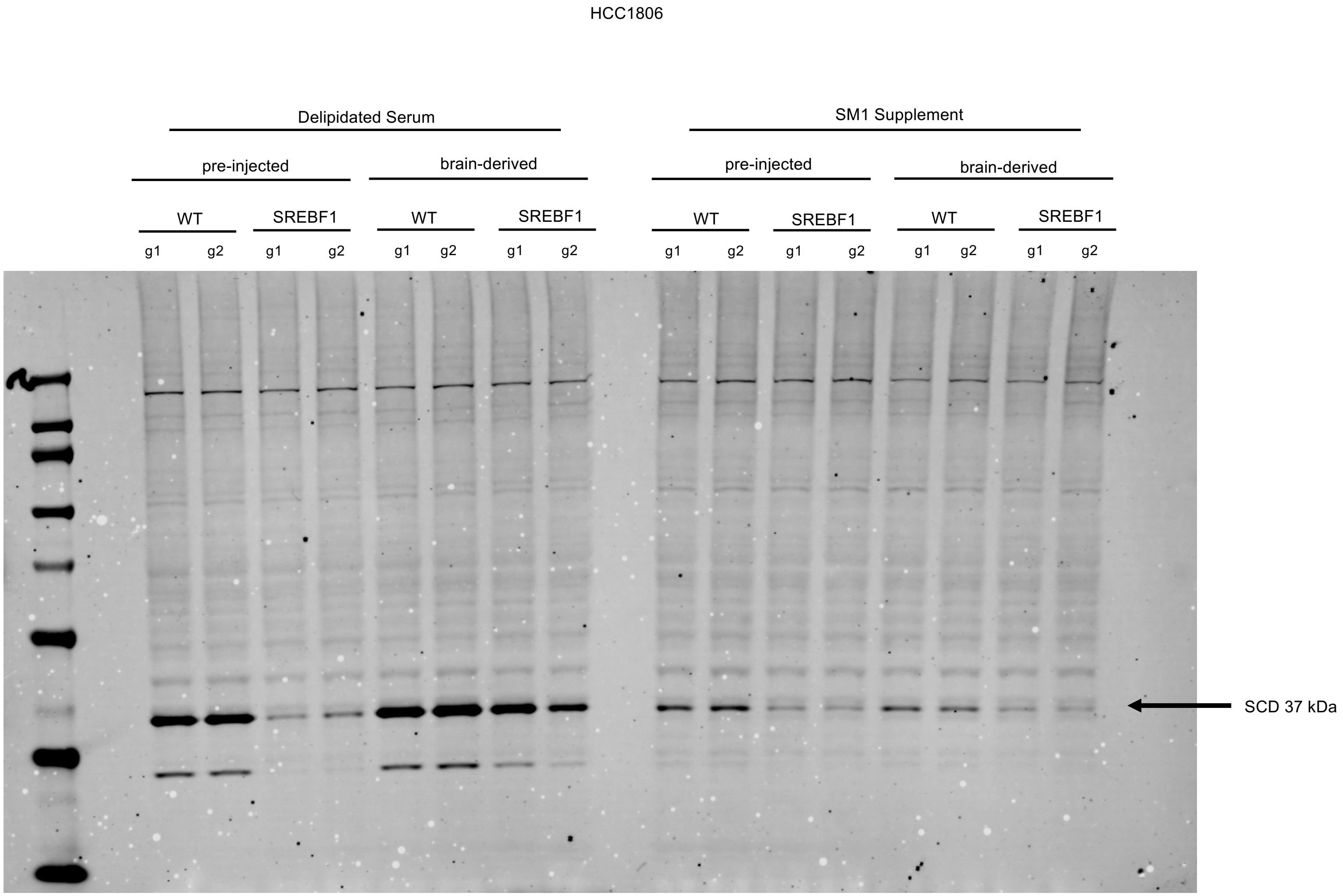
HCC1806
Delipidated Serum
SM1 Supplement
pre-injected
brain-derived
pre-injected
brain-derived
WT
SREBF1
WT
SREBF1
WT
SREBF1
WT
SREBF1
g1 g2 g1 g2 g1 g2 g1 g2 g1 g2 g1 g2 g1 g2 g1 g2
SCD 37 kDa

ED Fig. 11h

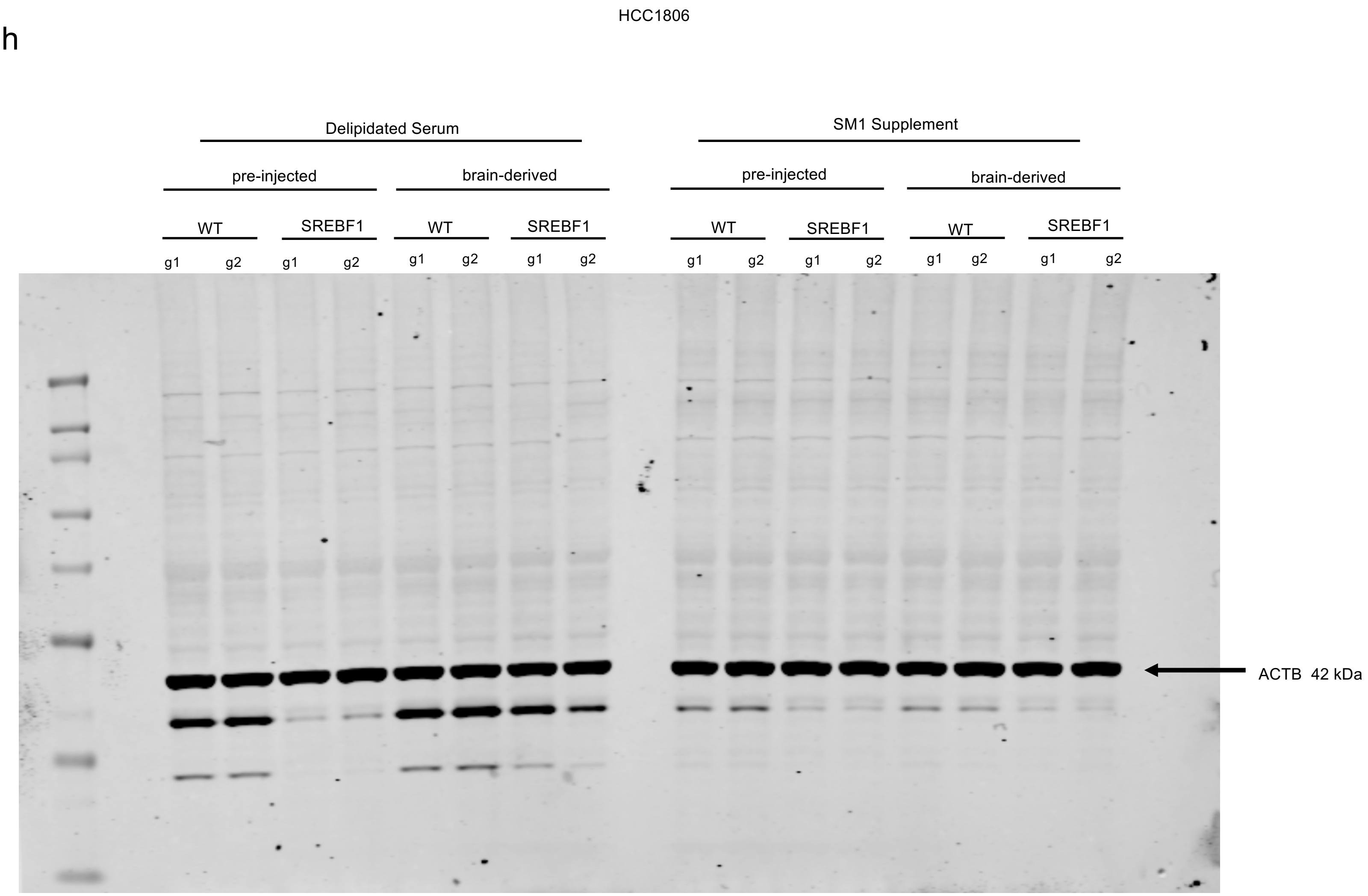

HCC1806
Delipidated Serum
SM1 Supplement
pre-injected
brain-derived
pre-injected
brain-derived
WT
SREBF1
WT
SREBF1
WT
SREBF1
WT
SREBF1
g1 g2 g1 g2 g1 g2 g1 g2 g1 g2 g1 g2 g1 g2 g1 g2
ACTB 42 kDa

ED Fig. 11h

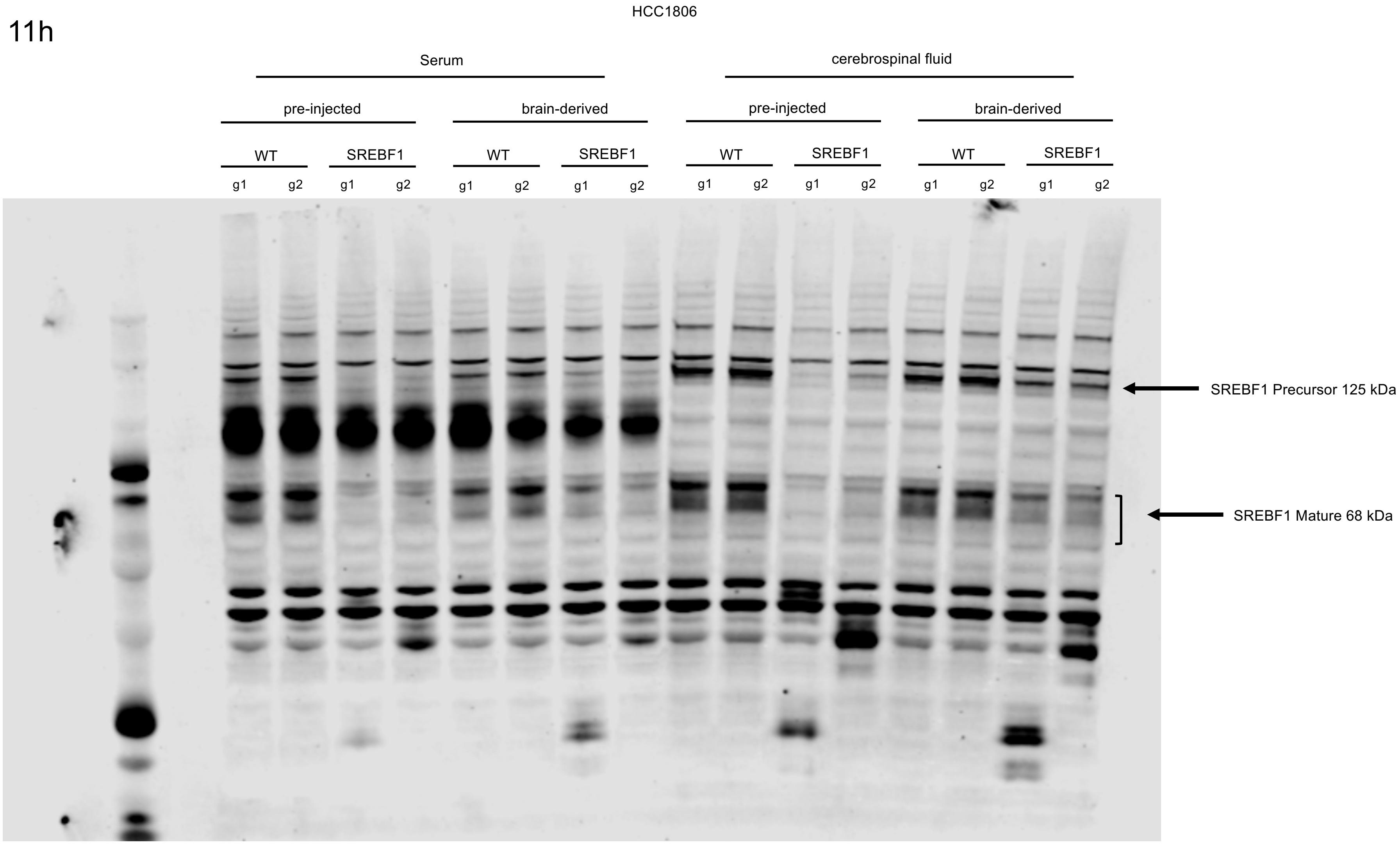
HCC1806
Serum
cerebrospinal fluid
pre-injected
brain-derived
pre-injected
brain-derived
WT
SREBF1
WT
SREBF1
WT
SREBF1
WT
SREBF1
g1 g2 g1 g2 g1 g2 g1 g2 g1 g2 g1 g2 g1 g2 g1 g2
SREBF1 Precursor 125 kDa
SREBF1 Mature 68 kDa

ED Fig. 11h

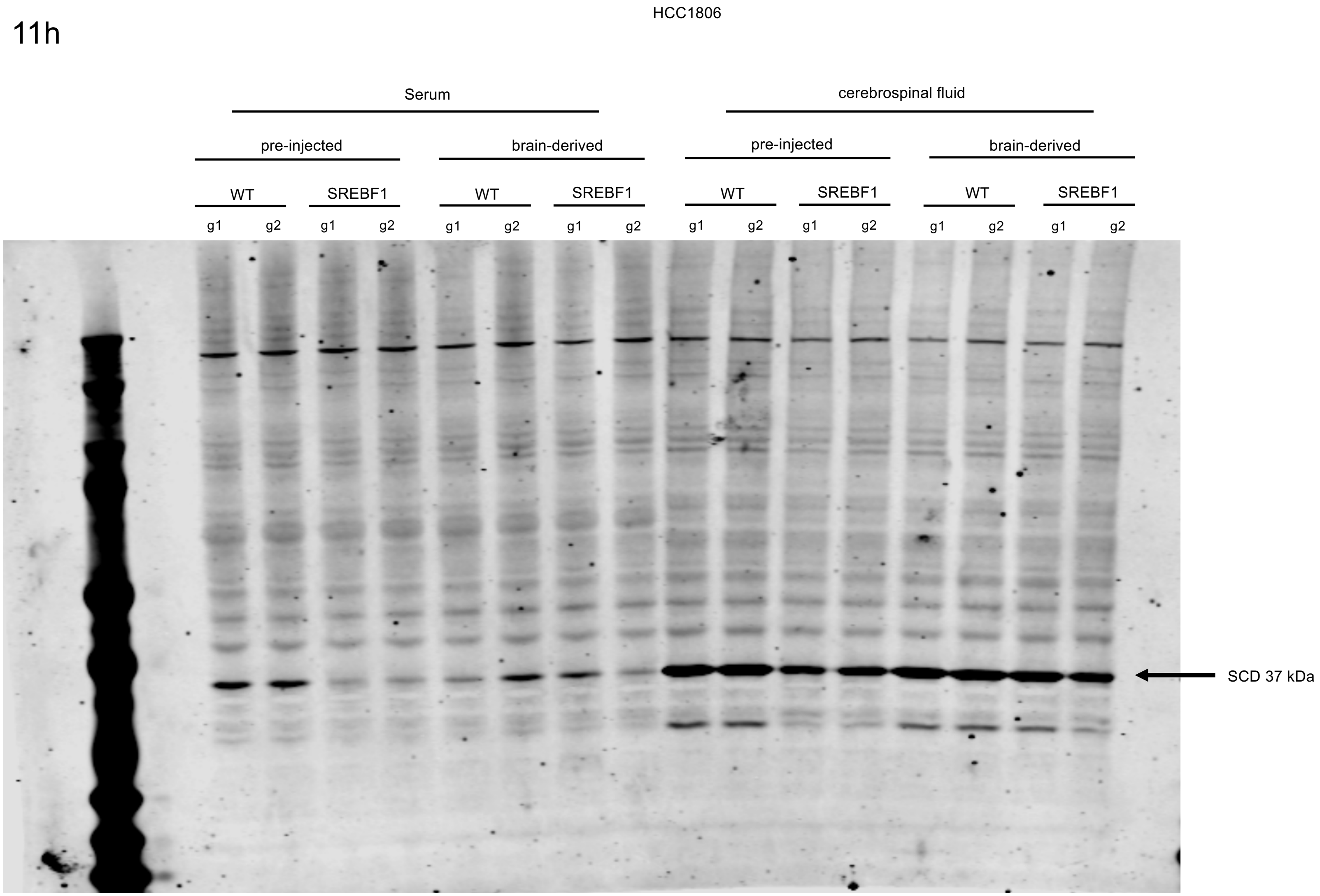
HCC1806
Serum
cerebrospinal fluid
pre-injected
brain-derived
pre-injected
brain-derived
WT
SREBF1
WT
SREBF1
WT
SREBF1
WT
SREBF1
g1 g2 g1 g2 g1 g2 g1 g2 g1 g2 g1 g2 g1 g2 g1 g2
SCD 37 kDa

ED Fig. 11h

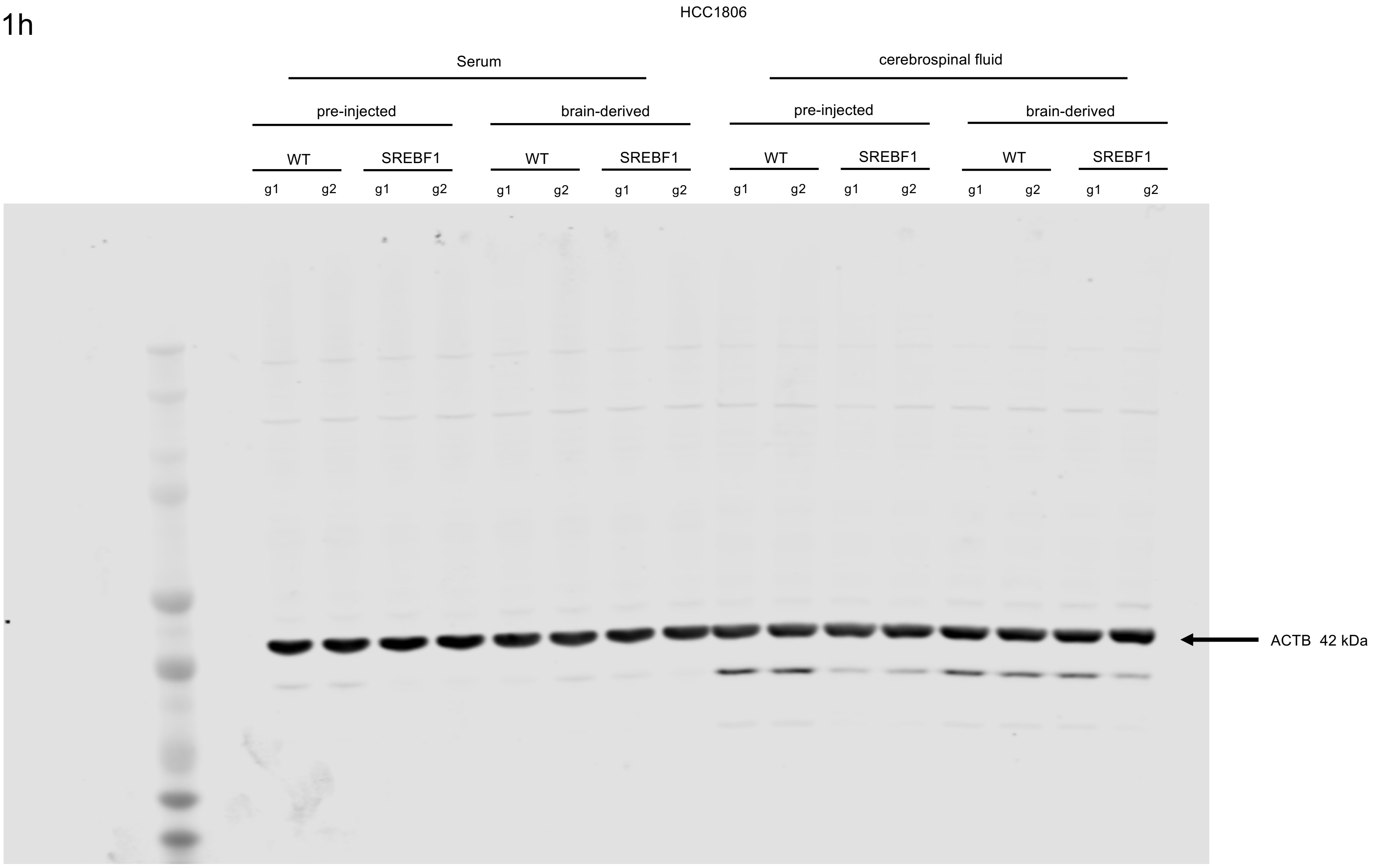
HCC1806
Serum
cerebrospinal fluid
pre-injected
brain-derived
pre-injected
brain-derived
WT
SREBF1
WT
SREBF1
WT
SREBF1
WT
SREBF1
g1 g2 g1 g2 g1 g2 g1 g2 g1 g2 g1 g2 g1 g2 g1 g2
ACTB 42 kDa
